# On a nonlinear hybrid method for multiscale analysis of a bearing‐capacity test of a real‐scale segmental tunnel ring

**DOI:** 10.1002/nag.2894

**Published:** 2019-02-04

**Authors:** Jiao‐Long Zhang, Herbert A. Mang, Xian Liu, Yong Yuan, Bernhard Pichler

**Affiliations:** ^1^ Institute for Mechanics of Materials and Structures TU Wien – Vienna University of Technology Vienna Austria; ^2^ College of Civil Engineering Tongji University Shanghai China

**Keywords:** discontinuities of static and kinematic variables, multilevel computation approach, nonlinear analysis, structural failure, transfer relations

## Abstract

A nonlinear hybrid method is developed for multiscale analysis of a bearing‐capacity test of a real‐scale segmental tunnel ring subjected to point loads. The structural analysis consists of two parts. Part I refers to modeling of bending‐induced tensile cracking of the segments, resulting from the external loading. The segments are subdivided into elements, according to the crack spacing. Each element is either intact or contains one central crack band, flanked by lateral undamaged domains. A multiscale model for tensile softening of concrete is used to describe the progressive deterioration of the crack bands. After iterative determination of their state of damage, the effective bending and extensional stiffnesses of the corresponding elements are quantified by means of Voigt‐Reuss‐Hill estimates. The effective stiffnesses are used for linear‐elastic simulations of the segmental tunnel ring. Part II refers to the relative rotation angles at the joints, which are estimated from monitoring data, using the Bernoulli‐Euler hypothesis. Since the validity of this hypothesis is questionable for neck‐like joints, the relative rotation angles are post‐processed such that they refer to rigid body displacements of the segments. The following conclusions are drawn: The presented approach yields good estimates of crack widths. Relative rotation angles at the joints mainly result in rigid body displacements of the segments, governing the convergences. Because realistic interface models are lacking, hybrid analysis based on displacement‐monitoring data allows for performing ultimate‐load analysis of segmental tunnel rings.

## 1 INTRODUCTION

The linings of tunnels, excavated by boring machines, consist of segmental rings. The individual segments are made of precast reinforced concrete. The interaction with the ground mass^1^, ^2^ and the resulting behavior of the joints between neighboring segments^3^, ^4^ govern the structural behavior of the lining. The mechanical behavior of the joints depends on their design, including the geometric layout of the initial segment‐to‐segment contact area^5^ and the use of bolts, connecting neighboring segments, either non‐prestressed or prestressed.^6^, ^7^ The behavior of the joints is nontrivial, because of (1) nonlinearities resulting from bending‐induced partial segment‐from‐segment separation,^8^, ^9^ (2) nonlinearities resulting from the material behavior, including crushing of concrete^5^, ^10^ and yielding of the steel bolts, 6, 7 and (3) the time‐dependent viscoelastic material behavior of concrete.^11^ The aim of the present paper is to consider *all* of these influence factors in the framework of analyzing a bearing‐capacity test of a segmental tunnel ring,^12^ *without the need to explicitly model* the nontrivial joint behavior. This will be achieved with the help of experimental data from monitoring of the displacement discontinuities at the joints during the real‐scale test. These data are used as input for the structural analysis. The analysis is based on the linear theory of slender circular arches.^13^

The first analytical models for structural analysis of segmental tunnel rings were simply based on closed rings, without explicit consideration of the joints, see the pioneering developments of Morgan^14^ and Wood.^15^ Lee and Gee^16^ analyzed several segmental tunnel rings, differing by the number of segments and the positions of the joints. They determined customized “reduction factors” for the stiffness of the continuous‐ring models, such that analytical structural analysis could be carried out with “equivalent” continuous tunnel rings. Explicit consideration of the joints was introduced by Lee et al.^17^ They calculated the relative rotation angles at the joints as linear functions of the internal forces transmitted across the joints, using the unit force method for determination of the internal forces and the displacements resulting from both the external loading *and* the relative rotation angles. Blom^18^ as well as El Nagger and Hinchberger^19^ simplified this mode of analysis. They argued that the relative rotation angles at the joints result in rigid body displacements of the segments. Thus, the present structural analysis of segmental tunnel rings typically consists of the following three steps:

1. Computation of the internal forces as occurring in a continuous linear‐elastic ring, subjected to the external loading.
2. Computation of the relative rotation angles at the joints as functions of the normal forces and the bending moments transmitted across the joints, making use of spring models.
3. “Correction” of the computed relative rotation angles such that they correlate with rigid body displacements of the segments, which do not activate internal forces.

This approach was validated for *regular service* loads and coefficients of lateral ground pressure around 0.9, see, e.g. Blom.^18^ The aim of the present paper is to analyze a *bearing‐capacity* test of a segmental tunnel ring, which was subjected to point loads, simulating a coefficient of lateral ground pressure as small as 0.65.^12^ This poses two main challenges:

1. The pronounced anisotropy of the external loading resulted in significant bending of the tested ring, which produced tensile cracking of the segments. This has raised the need for modeling bending‐induced tensile cracking of the segments.
2. The external loading was increased up to the bearing capacity of the ring, such that plastic hinges occurred at several joints during the test. This has raised the need for consideration of the nontrivial joint behavior, based on monitoring data that were recorded during the test.

The present paper refers to the extension of the linear hybrid method developed by Zhang et al.^13^ The term *linear* implies that the segments are considered to behave linearly elastic. Accordingly, their structural behavior can be described by means of transfer relations, representing analytical solutions of the *linear* theory of circular arches. The term *hybrid* implies that the structural simulations do not only use the external loading as input, but also the measured discontinuities that have developed at the joints during structural testing. With this modeling approach, Zhang et al.^13^ analyzed the first four load steps of the bearing‐capacity test of a real‐scale segmental tunnel ring, which was carried out by Liu et al.^12^ The remaining 36 load steps could not be considered, because bending‐induced tensile cracking of the segments was observed after load step 4. Consideration of all 40 load steps is the aim of the present paper.

The first central goal of the present work is to extend the described linear hybrid method towards consideration of the nonlinearities associated with bending‐induced tensile cracking of the segments. The second central goal is to organize the nonlinear hybrid method such that it follows the conceptual lines of Blom^18^ as well as El Nagger and Hinchberger,^19^ see above. The resulting nonlinear hybrid method will be applied to multiscale analysis of all 40 load steps of the bearing‐capacity test by Liu et al.^12^ In order to assess the suitability of the hybrid analysis, the simulated convergences of the tunnel ring will be compared with corresponding experimental measurements. Thereafter, two alternative simulations will be carried out. They refer to the following two items:

1. The assumption that the relative rotation angles at the joints are related to rigid body displacements will be checked. This will be accomplished by comparing the results from two structural simulations: (1) nonlinear hybrid analysis, based on relative rotation angles referring to rigid body displacements of the segments and (2) an alternative nonlinear hybrid analysis, based on relative rotation angles, which are estimated from structural monitoring data. However, they are not post‐processed in order to enforce a correlation with rigid body displacements of the segments.
2. The significance of accounting for bending‐induced tensile cracking of the segments will be assessed. This will be accomplished by comparing the results from two structural simulations: (1) nonlinear hybrid analysis accounting for tensile cracking and (2) linear hybrid analysis, disregarding tensile cracking.

The paper is organized as follows. Section 2 contains a brief description of the bearing‐capacity test of a real‐scale segmental tunnel ring, carried out by Liu et al.^12^ Section 3 is devoted to the development of the nonlinear hybrid method and its application to multiscale analysis of the test by Liu et al. Section 4 contains the discussion of the two central goals of the paper. Section 5 contains the conclusions drawn from the present investigation.

## 2 EXPERIMENTAL DATA

**Figure 1:**
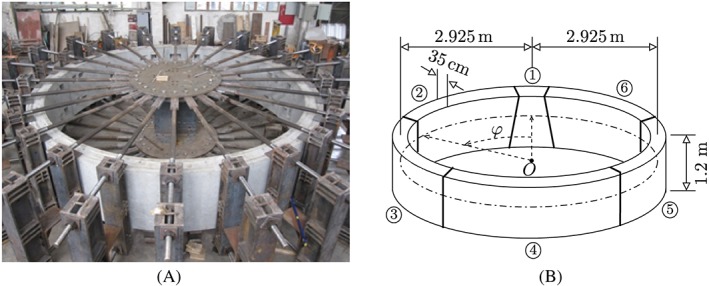
(A) Photo and (B) geometric dimensions of the analyzed segmental tunnel ring according to Liu et al.^12^ and Zhang et al.^13^ respectively. ①–⑥ refer to the number of the segments, and φ stands for the angular coordinate, measured from the center of the segment ① [Colour figure can be viewed at wileyonlinelibrary.com]

Inspired by other tests on segmental tunnel linings described in the open literature,^3^, ^18^ a bearing‐capacity test of a real‐scale segmental tunnel ring was recently carried out at Tongji University, Shanghai, see Liu et al.^12^ The radius *R* of the centerline of the ring was 2.925 m, see Figure 1. A polar coordinate system was used to describe the ring. The angular coordinate *φ* is measured from the crown.

**Figure 2:**
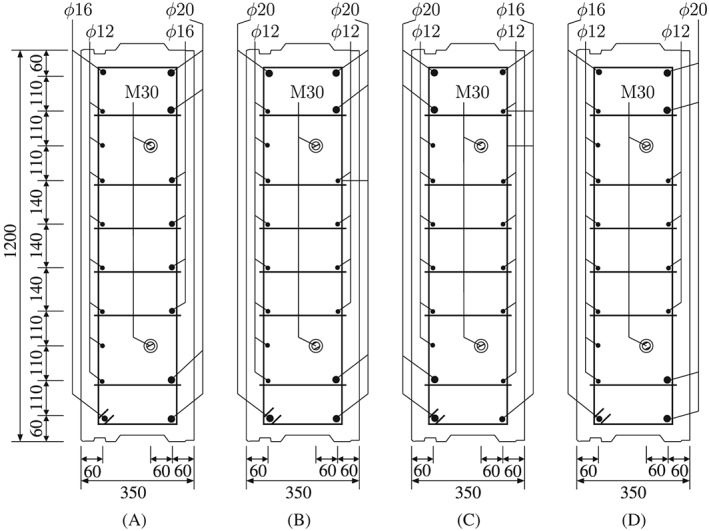
Cross‐sectional dimensions and sketch of the arrangement of the reinforcement: (A) segment ①, (B) segments ② and ⑥, (C) segments ③ and ⑤, and (D) segment ④; unit: mm

The ring consisted of six reinforced concrete segments. Their thickness, H, and their axial length, B, amounted to 35 cm and 1.2 m, respectively. As for the concrete, the maximum size of the aggregates, d_max_, was equal to 2 cm. The composition details of the concrete is listed in Table 1. The uniaxial compressive strength of concrete, f_c_, reached 28 days after production, amounted to 58 MPa. As for the reinforcement, Chinese hot‐rolled steel rebars with specification HRB 335 were used; see Figure 2 for the reinforcement drawings. Young's modulus, E_s_, and the yield stress of the reinforcement, f_y_, amounted to 210 GPa and 335 MPa, respectively. The concrete cover was 6 cm.

**Table 1:** Composition of the concrete used for the production of the segments

	Cement	Fly Ash	Slag	Water	Sand	Aggregates
Dosage, kg/m^3^	323	67	57	152	631	1169

The joints were positioned at angular coordinates φ_1_  =  8°, φ_2_  =  73°, φ_3_  =  138°, φ_4_  =  222°, φ_5_  =  287°, and φ_6_  =  352°. Neighboring segments were connected with two steel bolts each. The bolts had a diameter, d, amounting to 30 mm and a yield stress, f_b_, amounting to 400 MPa, see Figure 2.

**Figure 3:**
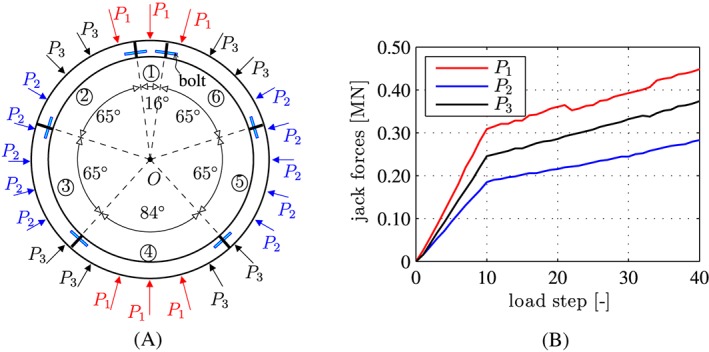
(A) Positions of the hydraulic jacks, imposing point loads onto the segmental tunnel ring, and (B) intensities of the jack forces prescribed during the bearing‐capacity test, see Liu et al.^12^ [Colour figure can be viewed at wileyonlinelibrary.com]

Compressive loading was imposed by three groups of altogether 24 hydraulic jacks, see Figure 3A. They simulated the action of non‐uniform ground pressure. Figure 3B shows the prescribed force intensities.

**Figure 4:**
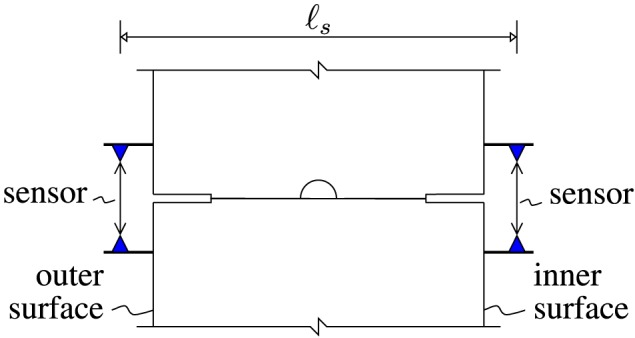
Positions of the displacement sensors measuring the discontinuities of the circumferential displacement across the inner and outer gaps of the joints; the sensor distance, ℓ_s_, amounted to 410 mm [Colour figure can be viewed at wileyonlinelibrary.com]

**Figure 5:**
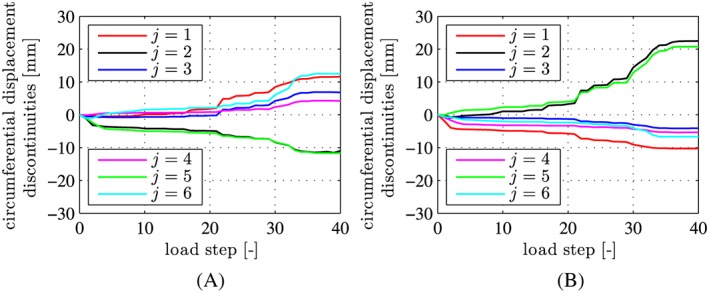
Data from structural monitoring: circumferential displacement discontinuities, measured across (A) the inner and (B) the outer gaps of the joints, see Liu et al.^12^; j  =  1,2,…,6 in the legends refer to the joints positioned at φ_j  =  1,2,…,6_  =  [8°,73°,138°,222°,287°,352°], respectively [Colour figure can be viewed at wileyonlinelibrary.com]

**Figure 6:**
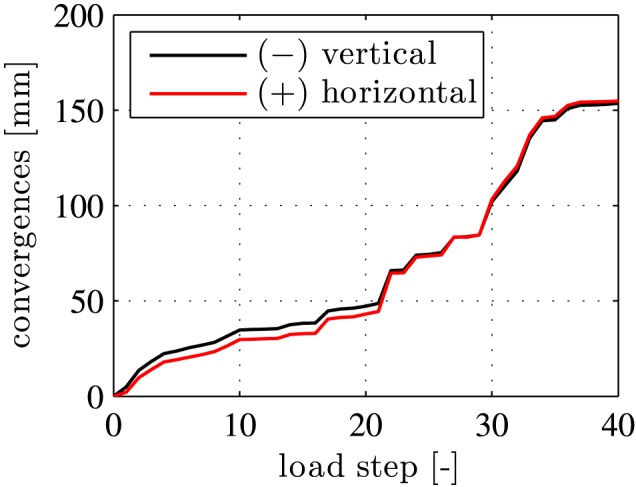
Data from structural monitoring: measured convergences (ovalization displacements); “+” and “−” refer to an increase and a decrease of the initial diameter, respectively, see Liu et al.^12^ [Colour figure can be viewed at wileyonlinelibrary.com]

The structural behavior was monitored carefully during the entire bearing‐capacity test. Displacement monitoring referred to circumferential displacement discontinuities across both the inner and the outer gaps of the joints (see Figures 4 and 5) and to the vertical and the horizontal convergences (see Figure 6). In addition, the occurrence of visible tensile cracking of concrete at the outer surface of the segments and of visible compressive crushing of concrete at the joints was documented. At the final load step, i.e. at load step 40, the widths of the bending‐induced tensile cracks was in the range of [0.05 mm, 0.10 mm], their spacing amounted to some 50 cm, and compressive crushing of concrete was observed at the joints at *φ*_1_  =  8°, *φ*_2_  =  73°, and *φ*_5_  =  287°. Load step 40 was very close to the bearing capacity of the tested segmental tunnel ring, because the convergences of the structure became so large that a kinematic mechanism began to develop.^12^ Notably, there were neither indications of yielding of the steel reinforcement of the segments nor of compressive crushing of the concrete *within* the segments.

## 3 MULTISCALE HYBRID ANALYSIS

### 3.1 Transfer relations

Structural analysis of the bearing‐capacity test is based on the following transfer relations, representing analytical solutions of the linear theory of slender circular arches, see Zhang et al.^13^
(1)
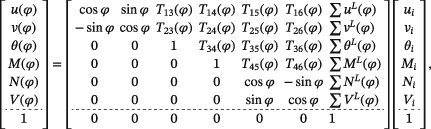
 where

(2) $$\begin{array}{ccc} T_{13}(\varphi ) & =R\;\sin\varphi \;,\;\;T_{14}(\varphi )=\frac{R^{2}}{EI}(\cos\varphi -1)\;, & \\ T_{15}(\varphi ) & =\frac{R}{EA}\frac{1}{2}\varphi \sin\varphi +\frac{R^{3}}{EI}\left(\frac{1}{2}\varphi \sin\varphi +\cos\varphi -1\right),\\ T_{16}(\varphi ) & =\frac{R}{EA}\left(\frac{1}{2}\varphi \cos\varphi -\frac{1}{2}\sin\varphi \right)+\frac{R^{3}}{EI}\left(\frac{1}{2}\varphi \cos\varphi -\frac{1}{2}\sin\varphi \right),\\ T_{23}(\varphi ) & =R\;(\cos\varphi -1)\;,\;\;T_{24}(\varphi )=\frac{R^{2}}{EI}(\varphi -\sin\varphi )\;,\\ T_{25}(\varphi ) & =\frac{R}{EA}\left(\frac{1}{2}\varphi \cos\varphi +\frac{1}{2}\sin\varphi \right)+\frac{R^{3}}{EI}\left(\varphi -\frac{3}{2}\sin\varphi +\frac{1}{2}\varphi \cos\varphi \right),\\ T_{26}(\varphi ) & =\frac{R}{EA}\left(-\frac{1}{2}\varphi \sin\varphi \right)+\frac{R^{3}}{EI}\left(1-\cos\varphi -\frac{1}{2}\varphi \sin\varphi \right),\\ T_{34}(\varphi ) & =-\frac{R}{EI}\varphi \;,\;\;T_{35}(\varphi )=\frac{R^{2}}{EI}(\sin\varphi -\varphi )\;,\;\;T_{36}(\varphi )=\frac{R^{2}}{EI}(\cos\varphi -1)\;,\\ T_{45}(\varphi ) & =R\;(1-\cos\varphi )\;,\;\;T_{46}(\varphi )=R\;\sin\varphi \;. \end{array}$$

In Equations 1 and 2, *R*, *E* *A*, and *E* *I* denote the radius of the axis of the arch, the extensional stiffness, and the bending stiffness, respectively. Further, *θ* stands for the cross‐sectional rotation, *u* and *v* denote the radial and the circumferential displacement, respectively, and *N*, *M*, and *V* stand for the axial force, the bending moment, and the shear force, respectively. The vector on the left‐hand side of Equation 1 contains the six kinematic and static variables, referring to the cross‐section at an arbitrary value of the angular coordinate *φ*. The vector on the right‐hand side of Equation 1 contains six integration constants. They represent three kinematic and static variables each, referring to the initial cross‐section (index “*i*”), i.e. to the circumferential position *φ*  =  0. The top‐left 6 × 6 submatrix of the transfer matrix in Equation 1 refers to the solution for an unloaded segmental tunnel ring.^13^ The summation symbols in the last column of the transfer matrix in Equation 1 indicate the superposition of so‐called load integrals (superscript “*L*”). The latter represent analytical solutions for radial point loads and relative rotation angles at the joints.

The load integrals for a radial point load *P*, imposed at position *φ*_*p*_, read as^13^

(3) $$u^{L}(\varphi )=\frac{1}{2}\left(\frac{PR}{EA}+\frac{PR^{3}}{EI}\right)\left[\sin(\varphi -\varphi_{p})-(\varphi -\varphi_{p})\cos(\varphi -\varphi_{p})\right]H(\varphi -\varphi_{p})\;,\;$$

(4) $$\begin{array}{ll} v^{L}(\varphi ) & =\frac{PR}{EA}\left[\frac{1}{2}(\varphi -\varphi_{p})\sin(\varphi -\varphi_{p})\right]H(\varphi -\varphi_{p})+\frac{PR^{3}}{EI}\left[\frac{1}{2}(\varphi -\varphi_{p})\sin(\varphi -\varphi_{p})+\cos(\varphi -\varphi_{p})-1\right]H(\varphi -\varphi_{p})\;, \end{array}$$

(5) $$\theta^{L}(\varphi )=\frac{PR^{2}}{EI}\left[1-\cos(\varphi -\varphi_{p})\right]H(\varphi -\varphi_{p})\;,$$

(6) $$M^{L}(\varphi )=-R\;P\sin(\varphi -\varphi_{p})H(\varphi -\varphi_{p})\;,$$

(7) $$N^{L}(\varphi )=P\sin(\varphi -\varphi_{p})H(\varphi -\varphi_{p})\;,$$

(8) $$V^{L}(\varphi )=-P\cos(\varphi -\varphi_{p})H(\varphi -\varphi_{p})\;,$$

where *H*(*φ* − *φ*_*j*_) stands for the Heaviside function. The load integrals for the relative rotation angle, Δ*θ*_*j*_, at the joint located at *φ*  =  *φ*_*j*_, read as^13^

(9) $$u^{L}(\varphi )=-R\Delta \theta_{j}\sin(\varphi -\varphi_{j})H(\varphi -\varphi_{j})\;,$$

(10) $$v^{L}(\varphi )=R\Delta \theta_{j}\left[1-\cos(\varphi -\varphi_{j})\right]H(\varphi -\varphi_{j})\;,$$

(11) $$\theta^{L}(\varphi )=\Delta \theta_{j}H(\varphi -\varphi_{j})\;,$$

(12) $$N^{L}(\varphi )=V^{L}(\varphi )=M^{L}(\varphi )=0\;.$$

The six integration constants are obtained as follows. Without loss of generality, the three integration constants, representing kinematic quantities, may be set equal to zero, i.e.

(13) $$u_{i}=v_{i}=\theta_{i}=0\;,$$

because they describe a rigid body displacement of the ring, see Zhang et al.^13^ Computation of the three integration constants, representing static quantities, is based on three continuity conditions of a *closed* ring. They read as

(14) θ(φ=0)=θ(φ=2π),

(15) u(φ=0)=u(φ=2π),

(16) v(φ=0)=v(φ=2π),

resulting in^13^, ^20^

(17) $$M_{i}=\frac{EI}{2R\pi \left(EAR^{2}+EI\right)}\left[2EAR\sum v^{L}(2\pi )+\left(3EAR^{2}+EI\right)\sum \theta^{L}(2\pi )\right]\;,\;$$

(18) $$N_{i}=-\frac{EIEA}{R\pi \left(EAR^{2}+EI\right)}\left[\sum v^{L}(2\pi )+R\sum \theta^{L}(2\pi )\right]\;,$$

(19) $$V_{i}=-\frac{EIEA}{R\pi \left(EAR^{2}+EI\right)}\sum u^{L}(2\pi )\;.$$

Equations (1), (2), (3), (4), (5), (6), (7), (8), (9), (10), (11), (12), (13), (14), (15), (16), (17), (18), (19) allow for analytical structural analysis of segmental tunnel rings, provided that the material behavior of the segments is linear elastic, the external loading is known, and the relative rotation angles at the joints were either measured (see Zhang et al.^13^) or computed by means of interface models (see Zhang et al.^20^).

### 3.2 Organization of the nonlinear hybrid analysis in form of two load cases

The aim of the following considerations is to carry out a hybrid analysis of the bearing‐capacity test described in Section 2, using the previously listed transfer relations. This involves two challenges: (1) extension of the linear transfer relations to consideration of bending‐induced tensile damage of the segments and (2) quantification of the relative rotation angles at the joints, considering their influence on the structural behavior. Conceptually, the proposed approach follows scientific work by Blom^18^ and by El Nagger and Hinchberger.^19^ They all assumed that the relative rotation angles at the joints result in *rigid body displacements* of the segments. This allows for subdividing hybrid analyses into two load cases. Load case I refers to the point loads, while the relative rotation angles are set equal to zero. Thus, the corresponding structural analysis may be interpreted as referring to a closed ring *without* joints, which is subjected to the point loads. This part of the hybrid analysis requires consideration of bending‐induced tensile cracking of the segments. Load case II refers to the relative rotation angles at the joints and to the rigid body displacements of the segments, caused by these relative rotations, while the point loads are set equal to zero. Finally, the two load cases will be superimposed. Such a superposition is admissible, even though the analysis of load case I is *nonlinear*, because (1) superposition of load case II only means addition of rigid body displacements and (2) equilibrium equations are formulated for the undeformed configuration.

**Figure 7:**
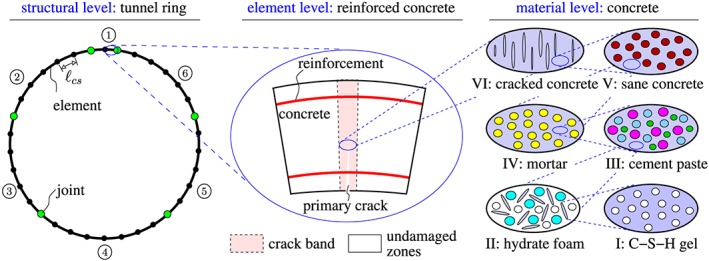
Multiscale organogram of a segmental tunnel ring [Colour figure can be viewed at wileyonlinelibrary.com]

The extension of the linear transfer relations to consideration of bending‐induced tensile damage of the segments is carried out in the framework of multiscale structural analysis, see Figure 7. At first, homogenization approaches of continuum micromechanics are employed to predict the stiffness and strength properties of the concrete of the segments. Subsequently, the ring is subdivided into 34 elements, see Figure 7. Their length, measured in the circumferential direction, is equal to the crack spacing, such that each element is either intact or contains one and only one crack band. In order to check whether or not bending‐induced tensile cracking occurs within the elements, both the bending moment and the normal force are averaged over the length of the elements. Based on the averaged quantities, a check is performed whether or not tensile cracking occurs. If it does, the element is subdivided into three zones: a central crack band, flanked by two undamaged lateral zones, see Figure 7. A multiscale model for tensile failure and softening of concrete is used to quantify the extensional stiffness and the bending stiffness of the crack band. Subsequently, the Voigt‐Reuss‐Hill average is used to quantify the average extensional stiffness and the average bending stiffness of the damaged element. Thus, the overall structural analysis is based on effective stiffnesses that are constant within the elements, while allowing these effective stiffnesses to be different for different elements.

Quantification of the relative rotation angles at the joints is based on the experimental data illustrated in Figure 5. First *estimates* of the relative rotation angles are computed, based on the assumption of a *linear* distribution of circumferential displacement jumps along the radial length of the joints, i.e. on the Bernoulli‐Euler hypothesis. In this context, it is noteworthy that joints represent structural necks. The corresponding stress concentrations invalidate the Bernoulli‐Euler hypothesis. This raises the need to improve the estimates of the relative rotation angles. The improvement is carried out at the *structural scale* of the segmental tunnel ring. In more detail, following scientific work by Blom^18^ and by El Nagger and Hinchberger,^19^ it is assumed that the relative rotation angles at the joints result in *rigid body displacements* of the segments.

### 3.3 Homogenization of the stiffness and the strength of the concrete used for production of the segments

The mean field homogenization methods of continuum micromechanics allow for estimating homogenized properties of microheterogeneous materials.^21^ They account for the hierarchical organization of the material, the shape, the volume fractions, and the material constants of the heterogeneities, as well as for their mutual interaction at different scales of observation. As for homogenization of the stiffness and strength of concrete, the models by Königsberger et al.^22^ and Hlobil et al.^23^ are used, see Figure 7 for the material organogram and Table 2 for the material constants of microstructural constituents of concrete. Notably, the bearing‐capacity test of Section 2 was carried out 28 days after casting of the reinforced concrete segments. The corresponding volume fractions of the material constituents of concrete are quantified, based on the initial composition of the concrete (see Table 1), and the maturity of the material, expressed in terms of the hydration degrees of cement, fly ash, and slag, respectively (see Appendix A for details).

**Table 2:** Material constants of the microstructural constituents of concrete

	Young's Modulus		Mass Density	Fracture Energy	
Phase	*E*, GPa	Poisson Ratio *ν*	*ρ*, g/cm^3^	*G*, J/m^2^	Ref.
Solid C‐S‐H	57.1	0.27	‐	1.72	Pellenq and Van Damme^24^
					and Bauchy et al.^25^
Pores	0	0	0	‐	Pichler et al.^26^
Clinker	139.9	0.3	3.15	‐	Velez et al.^27^
Fly ash	105	0.2	2.09	‐	Hlobil^28^
Slag	78	0.3	2.93	‐	Hlobil^28^
Sand	95.57	0.08	2.65	‐	Pichler and Hellmich^29^
Aggregates	49.93	0.3	2.65	‐	Pichler et al.^30^

Homogenization of the isotropic elastic stiffness and the uniaxial compressive strength of concrete is carried out, using the model by Königsberger et al.^22^ This delivers the following results:

(20) E=43.6GPa,

(21) ν=0.24,

(22) $$f_{c}=62.0\;\text{MPa}\;,$$

where *E*, *ν*, and *f*_*c*_ denote Young's modulus, Poisson ratio, and the uniaxial compressive strength of concrete. The model‐predicted compressive strength (see Equation 22) is quite close to the experimentally determined value of 58 MPa. This underlines the usefulness of the employed homogenization approach. In the interest of performing a multiscale structural analysis, model‐predicted values are used for structural analysis in the present paper.

**Figure 8:**
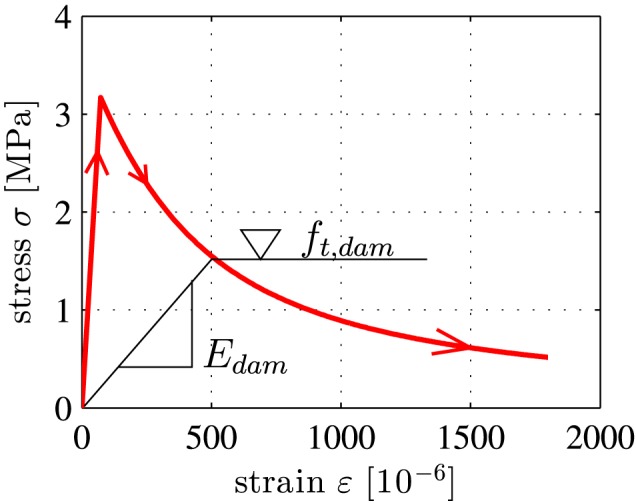
Constitutive model for concrete subjected to uniaxial tension [Colour figure can be viewed at wileyonlinelibrary.com]

Homogenization of the uniaxial tensile strength and of the tensile softening behavior is carried out, using the model by Hlobil et al.^23^ This delivers the following results:

(23) $$f_{t}=3.17\;\text{MPa}\;,$$

(24) $$f_{t,dam}(\omega )=\sqrt{\frac{0.402}{0.402+0.396\;\omega }}\;\cdot f_{t}\;,$$

(25) $$E_{dam}(\omega )=\frac{1}{1+5.02\;\omega }\;\cdot E\;,$$

where *f*_*t*_, *f*_*t*,*dam*_, and *E*_*dam*_ denote the uniaxial tensile strength of the concrete, its tensile strength during softening, and its Young's modulus during softening, respectively. Further, *ω* denotes the damage variable, which is equal to the crack density parameter by Budiansky and O'Connell.^31^ Equations 24 and 25 describe material softening, i.e. the decrease of the tensile strength and of Young's modulus with increasing damage. Equations 20 and (23), (24), (25) allow for computing stress‐strain diagrams for crack bands in concrete, see Figure 8. Unfortunately, the tensile strength was not determined experimentally. This renders a direct assessment of the model‐predicted value impossible.

### 3.4 Effective stiffness of segmental elements, damaged by bending‐induced tensile cracking

Linear‐elastic behavior of the segments are restricted to the first four load steps.^13^ Realistic simulation of the remaining load steps, i.e. load steps 5 to 40, requires consideration of bending‐induced tensile cracking of the segments. This rendered the subdivision of the segments into “simulation elements” necessary, whereby each element is considered either as intact or as containing one and only one crack band, see Figure 7. Therefore, the length of the elements, measured in the circumferential direction, is set equal to the crack spacing, *ℓ*_*cs*_, see Figure 7. The crack spacing is quantified, according to recommendations of the *fib* Model Code 32 as

(26) $$\ell_{cs}\approx 49\;\text{cm}\;.$$

This value agrees with the experimentally measured crack spacing, amounting to 50 cm, see Liu et al.^12^ Accordingly, the crown segment ① is subdivided into two elements of equal size, each of the lateral segments ②, ③, ⑤, and ⑥ is subdivided into six elements of equal size, and the bottom segment ④ is subdivided into eight elements of equal size, see Figure 7. Their undamaged extensional stiffnesses and bending stiffnesses as well as the length are listed in Table 3.

**Table 3:** Initial (undamaged) stiffnesses of the segments, lengths of the elements used for subdivision of the segments, and number of layers used for discretization of the crack band in the radial direction

	Extensional Stiffness	Bending Stiffness	Element Length	Number of
Segments	*E* *A*_*ini*_, MN	*E* *I*_*ini*_, MN m^2^	*ℓ*, m	Layers *n*_*l*_
①	18 260	186	0.41	18
② and ⑥	18 260	186	0.54	18
③ and ⑤	18 260	186	0.54	18
④	18 260	186	0.55	18

Structural analysis is organized in an incremental‐iterative fashion. Each one of the 40 load steps refers to one increment of the simulation. Within each increment, an iterative solution procedure is implemented. Each iteration step consists of an “elastic predictor‐step,” which is followed, if required, by “damage corrector‐steps.” Based on the results of the “elastic predictor‐step,” it is checked whether or not a “damage corrector‐step” is required. To this end, both the distribution of the bending moment *M*(*φ*) and the one of the normal force *N*(*φ*) are averaged along each of the 34 elements. The averaged quantities, ${\bar{M}}^{\left(e\right)}$ and ${\bar{N}}^{\left(e\right)}$, are obtained as

(27) $${\bar{M}}^{\left(e\right)}=\frac{1}{\psi_{f}^{\left(e\right)}}\int_{0}^{\psi_{f}^{\left(e\right)}}M(\psi )\;d\psi \;,$$

(28) $${\bar{N}}^{\left(e\right)}=\frac{1}{\psi_{f}^{\left(e\right)}}\int_{0}^{\psi_{f}^{\left(e\right)}}N(\psi )\;d\psi \;.$$

In Equations 27 and 28, the superscript *e* labels the number of the simulation element, i.e.  *e*  ∈  [1,2,…,34], and *ψ* denotes a *local* angular coordinate of the element, such that *ψ*  =  0 refers to the initial cross‐section and $\psi =\psi_{f}^{\left(e\right)}$ to the final cross‐section of the element. Insertion of *M*(*ψ*) and *N*(*ψ*) according to the fourth line and the fifth line of the transfer relations (1) into Equations 27 and 28 and integration of the resulting expression yield

(29) $${\bar{M}}^{\left(e\right)}=\frac{R}{\psi_{f}^{\left(e\right)}}\left\{V_{i}^{\left(e\right)}\left(1-\cos\psi_{f}^{\left(e\right)}\right)+N_{i}^{\left(e\right)}\left(\psi_{f}^{\left(e\right)}-\sin\psi_{f}^{\left(e\right)}\right)+P^{\left(e\right)}\left[1-\cos(\psi_{f}^{\left(e\right)}-\psi_{p}^{\left(e\right)})\right]\right\}+M_{i}^{\left(e\right)}\;,$$

(30) $${\bar{N}}^{\left(e\right)}=\frac{1}{\psi_{f}^{\left(e\right)}}\left\{V_{i}^{\left(e\right)}\left(\cos\psi_{f}^{\left(e\right)}-1\right)+N_{i}^{\left(e\right)}\sin\psi_{f}^{\left(e\right)}+P^{\left(e\right)}\left[\cos(\psi_{f}^{\left(e\right)}-\psi_{p}^{\left(e\right)})-1\right]\right\}.$$

In Equations 29 and 30, $M_{i}^{\left(e\right)}$, $N_{i}^{\left(e\right)}$, and $V_{i}^{\left(e\right)}$ denote the bending moment, the normal force, and the shear force at the initial cross‐section of the element, and *P* ^(*e*)^ stands for the point load imposed at the local angular position $\psi_{p}^{\left(e\right)}$. The element remains undamaged as long as the normal strains in the circumferential direction are smaller than or equal to the elastic limit:

(31) $$\varepsilon =\frac{{\bar{N}}^{\left(e\right)}}{EA_{ini}^{\left(e\right)}}+\frac{{\bar{M}}^{\left(e\right)}}{EI_{ini}^{\left(e\right)}}(r-R)\leq \frac{f_{t}}{E}\;\forall \;r\in \left[R-\frac{H}{2}\;,\;R+\frac{H}{2}\right],$$

where $EA_{ini}^{\left(e\right)}$ and $EI_{ini}^{\left(e\right)}$ denote the initial (undamaged) values of the extensional stiffness and of the bending stiffness, respectively, see Table 3. If the condition (31) turns out to be violated, damage is assumed to have started, resulting in a crack band (index *c* *b* *d*), which requires “damage corrector‐steps.”

**Figure 9:**
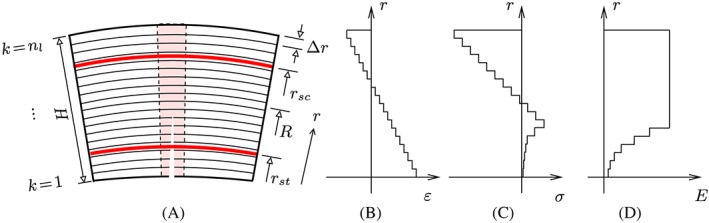
Modeling of segmental elements, damaged by bending‐induced tensile cracking: (A) subdivision of the element into concrete layers and layer‐wise constant values of (B) the normal strains in the circumferential direction, (C) the normal stresses in the circumferential direction, and (D) Young's modulus of concrete [Colour figure can be viewed at wileyonlinelibrary.com]

The typical width of a crack band in the concrete, *ℓ*_*cbd*_, measured normal to the crack planes, amounts to three times the maximum aggregate size,^33^ i.e. 

(32) $$\ell_{cbd}=3\;d_{max}\;,$$

see also Figures 7 and 9A. A damaged element is subdivided into one central crack band flanked by two undamaged lateral zones, see Figure 7. The structural stiffnesses *E* *A* and *E* *I* of the undamaged lateral zones remain equal to their initial values, *E* *A*_*ini*_ and *E* *I*_*ini*_, while those of the crack band decrease with increasing damage.

Determination of the extensional stiffness, *E* *A*_*cbd*_, and of the bending stiffness, *E* *I*_*cbd*_, of the crack band is based on (1) the Bernoulli‐Euler hypothesis, (2) linear‐elastic behavior of steel both in tension and in compression, and (3) linear‐elastic behavior of concrete in compression as well as linear‐elastic, nonlinear‐softening of concrete in tension according to Equations 20 and 23 to 25, see also Figure 8. The Bernoulli‐Euler hypothesis implies that a *linear* distribution of the normal strain in the circumferential direction prevails inside every crack band. Thus,

(33) $$\varepsilon^{\left(e\right)}=\varepsilon_{cbd}^{\left(e\right)}+\kappa_{cbd}^{\left(e\right)}\;(r-R)\;,$$

where $\varepsilon_{cbd}^{\left(e\right)}$ and $\kappa_{cbd}^{\left(e\right)}$ stand for the normal strain in the circumferential direction at the centerline of the crack band and for the change of curvature of this line, respectively. Both $\varepsilon_{cbd}^{\left(e\right)}$ and $\kappa_{cbd}^{\left(e\right)}$ are unknown and will be identified such that the stress resultants of the crack band,

(34) $${\bar{M}}^{\left(e\right)}=\int_{A_{cbd}}\sigma \cdot (r-R)\;dA\;,$$

(35) $${\bar{N}}^{\left(e\right)}=\int_{A_{cbd}}\sigma \;dA\;,$$

are equal to the average bending moment and the average normal force of the element, as computed in the structural simulation of the entire ring, see Equations 29 and 30. In this simulation, the crack band is subdivided, in the radial direction, into *n*_*l*_ (see Table 3) layers with a thickness amounting to Δ*r*, see Figure 9A. In each layer, the normal strain in the circumferential direction is considered to be constant, see Figure 9B. Thus, the strain in the *k* ^th^ layer is given as

(36) $$\varepsilon_{k}^{\left(e\right)}=\varepsilon_{cbd}^{\left(e\right)}+\kappa_{cbd}^{\left(e\right)}\;(r_{k}-R)\;,$$

with *r*_*k*_ denoting the radial coordinate of the midline of layer *k*. The layer‐wise constant strains are related to layer‐wise constant stresses and constant Young's moduli of concrete. They are based on Hooke's law as long as compressive strains are concerned and otherwise on linear‐elastic, nonlinear‐softening behavior according to Equations 20 and (23), (24), (25), see also Figure 8. This results in a “nonlinear” step‐wise distribution of the tensile normal stresses of concrete, see Figure 9C. As for the reinforcement bars, the strains follow from inserting their radial coordinates, *r*_*sc*_ and *r*_*st*_ (see Figure 9B), into Equation 33. The subscripts “*s* *c*” and “*s* *t*” stand for “steel in compression” and “steel in tension,” respectively. Multiplication of the result by Young's modulus of steel, *E*_*s*_, gives

(37) $$\sigma_{sc}^{\left(e\right)}=E_{s}\;\left[\varepsilon_{cbd}^{\left(e\right)}+\kappa_{cbd}^{\left(e\right)}\;(r_{sc}-R)\right]\;,$$

(38) $$\sigma_{st}^{\left(e\right)}=E_{s}\;\left[\varepsilon_{cbd}^{\left(e\right)}+\kappa_{cbd}^{\left(e\right)}\;(r_{st}-R)\right]\;.$$

Because of the layer‐wise constant stresses, the integrals in Equations 34 and 35 can be expressed in form of the following sums:

(39) $${\bar{M}}^{\left(e\right)}=\sum_{k=1}^{n_{l}}\sigma_{k}^{\left(e\right)}\;\Delta r\;B(r_{k}-R)+\sigma_{sc}^{\left(e\right)}A_{sc}(r_{sc}-R)+\sigma_{st}^{\left(e\right)}A_{st}(r_{st}-R)\;,$$

(40) $${\bar{N}}^{\left(e\right)}=\sum_{k=1}^{n_{l}}\sigma_{k}^{\left(e\right)}\;\Delta r\;B+\sigma_{sc}^{\left(e\right)}A_{sc}+\sigma_{st}^{\left(e\right)}A_{st}\;,$$

where *A*_*sc*_ and *A*_*st*_ denote the cross‐sectional area of the reinforcement in compression and tension, respectively. Equating the expressions on the right‐hand sides of (29) and (39) and of (30) and (40) delivers two equations for the two unknowns $\varepsilon_{cbd}^{\left(e\right)}$ and $\kappa_{cbd}^{\left(e\right)}$. They are solved numerically by the relaxation method.

Thereafter, the extensional stiffness $EA_{cbd}^{\left(e\right)}$ and the bending stiffness $EI_{cbd}^{\left(e\right)}$ of the crack band are computed as described in the following. The general definitions of the stiffness quantities read as

(41) $$EA_{cbd}=\int_{A_{cbd}}E\;dA\;$$

and

(42) $$EI_{cbd}=\int_{A_{cbd}}E\cdot {\left(r-R\right)}^{2}\;dA\;.$$

Consideration of linear‐elastic, nonlinear‐softening behavior of concrete in tension leads to layer‐wise constant Young's moduli of concrete, see Figure 9D. Thus, the integrals of Equations 41 and 42 can be expressed in the form of the following sums:

(43) $$EA_{cbd}^{\left(e\right)}=\sum_{k=1}^{n_{l}}E_{k}^{\left(e\right)}\;\Delta r\;B+E_{s}\;A_{sc}+E_{s}\;A_{st}\;,$$

(44) $$EI_{cbd}^{\left(e\right)}=\sum_{k=1}^{n_{l}}E_{k}^{\left(e\right)}\;\Delta r\;B{\left(r_{k}-R_{cbd}^{\left(e\right)}\right)}^{2}+E_{s}\;A_{sc}{\left(r_{sc}-R_{cbd}^{\left(e\right)}\right)}^{2}+E_{s}\;A_{st}{\left(r_{st}-R_{cbd}^{\left(e\right)}\right)}^{2},\;$$

where $R_{cbd}^{\left(e\right)}$ denotes the radial coordinate of the neutral axis of the crack band. It is computed as follows:

(45) $$R_{cbd}^{\left(e\right)}=\frac{1}{EA_{cbd}}\left(\sum_{k=1}^{n_{l}}E_{k}^{\left(e\right)}\;r_{k}\;\Delta r\;B+E_{s}\;r_{sc}\;A_{sc}+E_{s}\;r_{st}\;A_{st}\right).$$

The next task is to quantify the effective extensional and the bending stiffness, ${\bar{EA}}^{\left(e\right)}$ and ${\bar{EI}}^{\left(e\right)}$, respectively, of a damaged simulation element. These quantities are estimated based on the Voigt‐Reuss‐Hill average,^34^ which is defined as the arithmetic mean of the upper bound of the stiffnesses according to Voigt and of their lower bound according to Reuss:

(46) $${\bar{EA}}^{\left(e\right)}=\frac{1}{2}\left[f_{cbd}^{\left(e\right)}EA_{cbd}^{\left(e\right)}+(1-f_{cbd}^{\left(e\right)})EA_{ini}+{\left(\frac{f_{cbd}^{\left(e\right)}}{EA_{cbd}^{\left(e\right)}}+\frac{1-f_{cbd}^{\left(e\right)}}{EA_{ini}}\right)}^{-1\;}\right],\;$$

(47) $${\bar{EI}}^{\left(e\right)}=\frac{1}{2}\left[f_{cbd}^{\left(e\right)}EI_{cbd}^{\left(e\right)}+(1-f_{cbd}^{\left(e\right)})EI_{ini}+{\left(\frac{f_{cbd}^{\left(e\right)}}{EI_{cbd}^{\left(e\right)}}+\frac{1-f_{cbd}^{\left(e\right)}}{EI_{ini}}\right)}^{-1\;}\right].$$

In Equations 46 and 47, $f_{cbd}^{\left(e\right)}$ stands for the volume fraction of the crack band relative to the total volume of the element, such that $\left(1-f_{cbd}^{\left(e\right)}\right)$ is the volume fraction of the intact lateral parts of the element relative to its total volume. Denoting the volume of the element as *V* ^(*e*)^ and its length in the circumferential direction as *ℓ* ^(*e*)^, the former volume fraction follows as

(48) $$f_{cbd}^{\left(e\right)}=\frac{V_{cbd}^{\left(e\right)}}{V^{\left(e\right)}}=\frac{\ell_{cbd}}{\ell^{\left(e\right)}}=\frac{3\;d_{max}}{\ell^{\left(e\right)}}\;.$$

In the described model of the segmental elements, tensile softening of concrete is considered, while linear elasticity was assumed for the steel reinforcement and for concrete in compression. This assumption agrees with experimental observations by Liu et al.^12^ Still, it is interesting to check the stresses of the reinforcement and of concrete in compression, based on the results from structural hybrid analysis. The latter is nonlinear and, thus, requires an incremental‐iterative solution procedure.

### 3.5 Load case I: structural analysis, considering point loads

Following the organization of the hybrid analysis of the bearing‐capacity tests as outlined in Section 3.2, in load case I, the point loads, illustrated in Figure 3, are considered. The relative rotation angles at the joints are set equal to zero. The segmental ring is subdivided into 34 elements. Each of them is characterized by a constant extensional stiffness and a constant bending stiffness. Thus, the structural analysis involves 34 transfer matrices. The first one describes the structural behavior of the first element, extending from the crown to the interface between elements 1 and 2. The second transfer matrix describes the second element, extending from the interface between elements 1 and 2 to that between elements 2 and 3, and so on. Thus, a product of 34 transfer matrices describes the relation between the static and the kinematic variables referring to the *initial* cross‐section and the ones referring to the *final* cross‐section. This allows for formulation of the three continuity conditions (14) to (16) and, thus, for identification of the three integration constants, representing static variables at the crown of the ring, see Equations (17), (18), (19).

Structural analysis starts with load step 1. The initial values of the extensional stiffnesses and the bending stiffnesses are listed in Table 3. Once the simulation results indicate the onset of tensile cracking of one of the segments, see condition (31), “damage corrector‐steps” are carried out in an iterative fashion. At the beginning of each iteration step, the effective stiffnesses of the damaged elements are computed as described in Section 3.4. Thereafter, it must be ensured that damage can only increase, as is expected for progressive loading of the structure. In other words, the analysis must not admit spurious “healing” of segments in case of small unloading steps, see, e.g. the small reduction of the external forces *P*_1_ from load steps 21 to 22, shown in Figure 3B. This requirement is considered as follows:

• Computed values of ${\bar{EA}}^{\left(e\right)}$ and ${\bar{EI}}^{\left(e\right)}$ are accepted only if they are *smaller* than the values obtained at the *end* of the previous load increment.
• If the computed values of ${\bar{EA}}^{\left(e\right)}$ and ${\bar{EI}}^{\left(e\right)}$ are *larger* than the values obtained at the end of the previous load increment, unloading will take place. This implies that damage remains constant. Thus, the values of ${\bar{EA}}^{\left(e\right)}$ and ${\bar{EI}}^{\left(e\right)}$, obtained at the end of the previous load increment, are considered for further analysis.

Once the updated stiffnesses are identified, they are assigned to the corresponding simulation elements. This marks the end of the iteration step.

The next iteration step starts with a structural simulation of the entire segmental tunnel ring. This is followed by calculating the element‐wise average bending moments and normal forces according to Equations 29 and 30 and checking whether or not the new values are consistent with the damage states at the end of the previous iteration step. In case of inconsistencies, further “damage corrector‐steps” are carried out as described above. This iterative procedure is repeated until the averaged bending moments and normal forces at the end of two successive iteration steps do not change any more. In that case, the structural analysis continues with the next load step.

### 3.6 Results from nonlinear multiscale analysis: internal forces and deterioration of the segmental tunnel ring

The results of load case I refer to *final results* from the nonlinear hybrid analysis of the bearing‐capacity tests of Section 2, as far as the internal forces and the state of damage both of the segments and the joints are concerned. In this context, it is noteworthy that load case II refers to rigid body displacements only, i.e. it does not contribute to the internal forces and to the state of damage of the structure.

**Figure 10:**
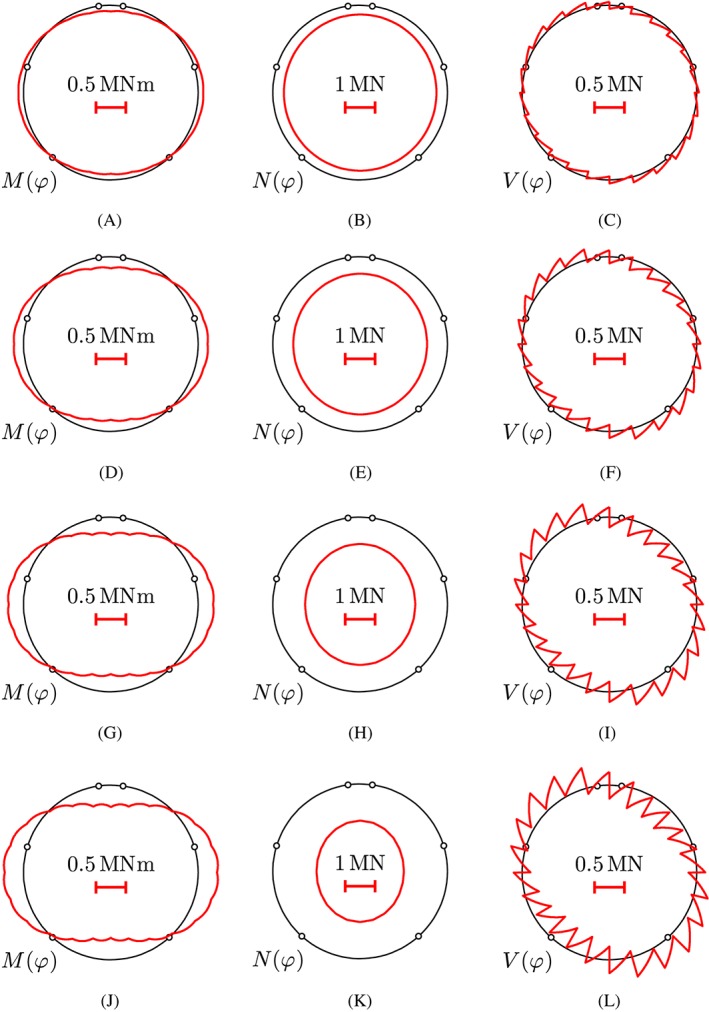
Results from load case I, “point loads”: distributions of internal forces; the first line of the illustrations refers to load step 4, the second line to load step 7, the third line to load step 16, and the fourth line to load step 40, associated with 25%, 50%, 75%, and 100% of the bearing capacity. The red graphs located inside/outside of the axis of the segmental ring refer to negative/positive internal forces [Colour figure can be viewed at wileyonlinelibrary.com]

The discussion of the results from load case I starts with the internal force distributions at the level of the entire segmental tunnel ring. The computed bending moments, normal forces, and shear forces are symmetric/antisymmetric with respect to the “vertical” axis passing through the center and the crown of the segmental tunnel ring, see Figure 10. At the crown and the bottom of the ring, the point loads activate negative bending moments, resulting in tensile stresses at the inner surface of the segments. In the lateral regions, positive bending moments prevail, resulting in tensile stresses at the outer surface of the segments. The normal forces in the top and bottom regions are slightly larger than those in the lateral regions, as expected from the anisotropic external loading, see Figure 3. The shear forces show saw‐tooth distributions, whereby the jumps are equal to the external point loads, see Equation 8.

**Figure 11:**
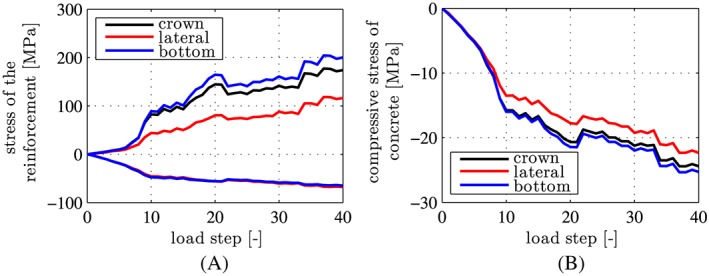
Results from load case I, “point loads”: extreme values of the computed stresses, transferred by the segments in the top element #1, the lateral element #9, and the bottom element #17, as a function of the load step: (A) axial stresses of the steel rebars, (B) compressive normal stresses of concrete, acting in the circumferential direction [Colour figure can be viewed at wileyonlinelibrary.com]

**Figure 12:**
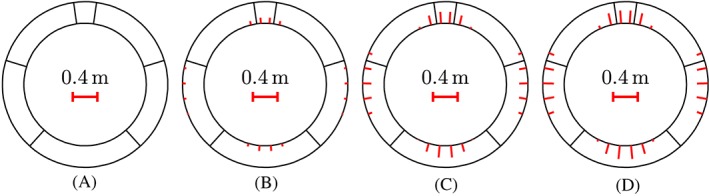
Depths of cracks resulting from load case I, “point loads”: (A) load step 4, (B) load step 7, (C) load step 16, and (D) load step 40, associated with 25%, 50%, 75%, and 100% of the bearing capacity [Colour figure can be viewed at wileyonlinelibrary.com]

A comparison of the computed axial stresses of the reinforcement bars with the yield stress of steel, *f*_*y*_  =  335 MPa, underlines the validity of the assumption of linear‐elastic behavior of steel, all the way up to the bearing capacity of the ring, see Figure 11A. By analogy, the computed extreme values of the compressive normal stresses in the circumferential direction, experienced by the concrete of the segments, are always smaller than the uniaxial compressive strength of the material, *f*_*c*_  =  62 MPa, see Figure 11B. Thus, also, the assumption of linear‐elastic behavior of concrete under compression is valid. Bending‐induced tensile cracks are distributed around the ring, except for regions around zero positions of the bending moment, as follows from a comparison of the crack pattern (see Figure 12) with the corresponding distribution of the bending moments (see Figure 10A, D, G, J). The depth of the cracks on the outer surface of the lateral region is smaller than that on the inner surface of the top and the bottom region, see Figure 12. The largest depth of the cracks is about 60% of the thickness of the segments. In order to calculate the widths of the cracks at the surface of the segments, the computed axial strain in the circumferential direction (see Equation 36) evaluated at the cracked surface of the corresponding crack band (positioned at *r*  =  *r*_*t*_) is multiplied by the size of this zone, equal to three times the maximum aggregate size, see Equation 32. This gives

(49) $$w_{c}^{\left(e\right)}=3\;d_{max}\;\left[\varepsilon_{cbd}^{\left(e\right)}+\kappa_{cbd}^{\left(e\right)}\;(r_{t}-R)\right]\;.$$

**Figure 13:**
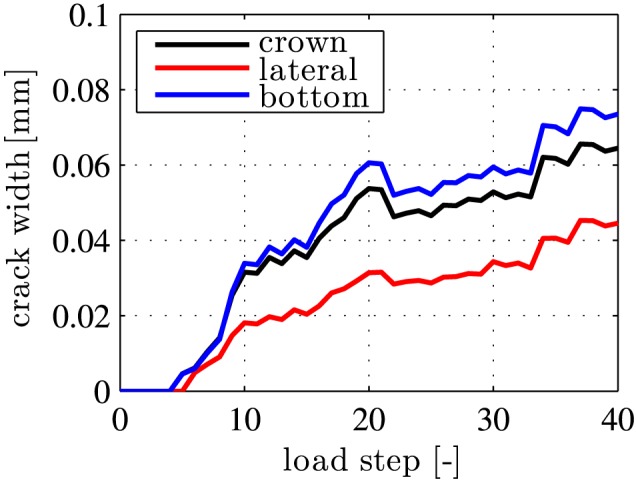
Results from load case I, “point loads”: crack widths at the surface of the segments in the top element #1, the lateral element #9, and the bottom element #17, as a function of the load step [Colour figure can be viewed at wileyonlinelibrary.com]

The local extreme values of the crack widths are obtained in the lateral regions and at the bottom, see Figure 13B. At the final load step, the maximum crack widths are within the interval [0.05 mm, 0.10 mm]. This is consistent with the independent experimental observations, see Section 2.

**Figure 14:**
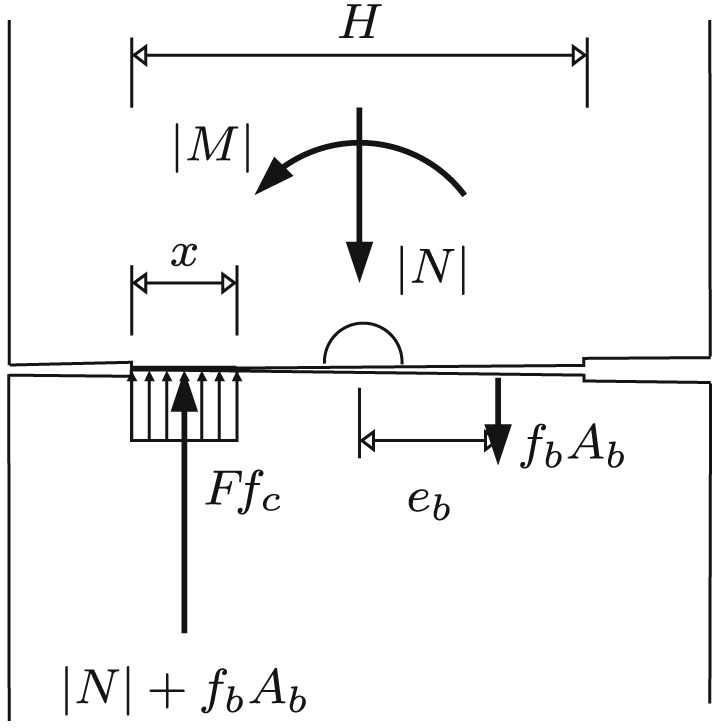
Stress distribution related to the first (“inner”) failure envelope

Next, the behavior of the six joints is described. In the test, they experienced a severe deterioration. Some of them even became plastic hinges. This has provided the motivation to compute *M*‐*N* interaction diagrams, analogous to the strategy proposed for concrete hinges by Kalliauer et al.^35^ The present analysis is based on the following *two* types of failure envelopes:

1. The first failure envelope is calculated based on the *initial* contact area of the segments (see Figure 14). The initial full‐face contact is lost because of the bending moments transmitted across the joints. In addition, the first failure envelope is based on tensile yielding of the steel bolts crossing the joint and on crushing of concrete at a compressive stress amounting to *F* times the uniaxial compressive strength *f*_*c*_. Thereby, *F* denotes a triaxial‐to‐uniaxial strength ratio, which accounts for the fact that stress concentrations in the neck‐like regions of the joints activate three‐dimensional compressive stress states, which result in confinement and, thus, in an increase of the compressive strength. Mathematical details regarding the first failure envelope are given in Appendix B.1.

Notably, the initial contact area at the joints is smaller than the cross‐sectional area of the segments, because the outermost and innermost regions of neighboring segments are initially separated by a gap, see Figure 4. Once a plastic hinge develops, such as described by the first failure envelope, the gap adjacent to the compressed side of the joint will close. Consequently, additional compressive stresses will be transmitted across the joint. In other words, after the development of a plastic hinge, as described by the first failure envelope, the behavior of the joint will become stiffer, once the described gap starts to close.

**Figure 15:**
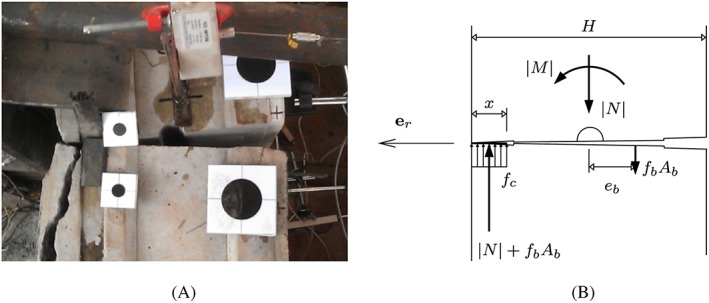
(A) Joint of the analyzed segmental tunnel ring at the ultimate limit state, according to Liu et al.,^12^ and (B) corresponding stress distribution, related to the second (“outer”) failure envelope [Colour figure can be viewed at wileyonlinelibrary.com]

2. The second failure envelope takes into account that the initial gap between two neighboring segments is completely closed at the compressive side, such that the uniaxial compressive strength is reached (see Figure 15). Thus, the second failure envelope refers to the bearing capacity of the joints. Mathematical details are given in Appendix B.2.

**Figure 16:**
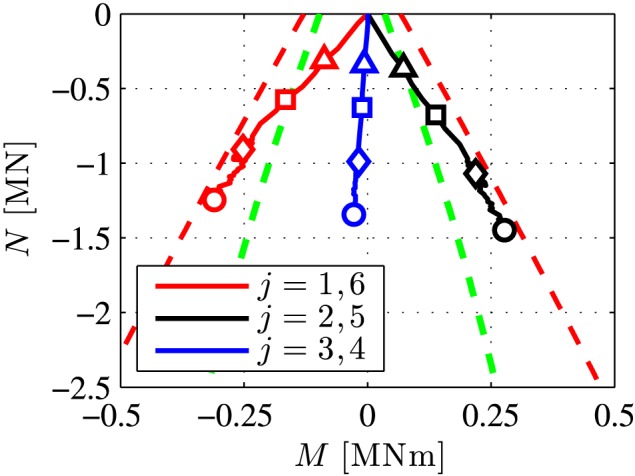
Comparison of the failure envelopes of the joints according to Equations B4 (see the green dashed line) and B7 (see the red dashed line), with the “M‐N loading paths” computed in load case I (see the red, black, and blue solid lines for the paths of the two top, lateral, and bottom joints, respectively); the triangles, squares, diamonds, and circles label results from load steps 4, 7, 16, and 40, associated with 25%, 50%, 75%, and 100% of the bearing capacity [Colour figure can be viewed at wileyonlinelibrary.com]

The predictive capabilities of the two types of failure envelopes are assessed by means of comparing them with the “*M*‐*N* loading paths” computed in load case I (see Figure 16). The first (“inner”) failure envelope is reached at the two lateral joints already at load step 4, and at the two top joints at load step 7, i.e. at intensities of the external loading, which are significantly smaller than the bearing capacity of the entire ring structure. The second (“outer”) failure envelope slightly overestimates the bearing capacity of the joints, because the computed “*M*‐*N* loading paths” approach the envelope without reaching it. This suggests that the final contact along the initial gap in the outer/innermost regions of the segments, obtained in the real‐scale experiment, was close, but not equal, to full‐face contact. Still, Figure 16 underlines that, starting with load step 4, some of the joints have developed plastic hinges. This provides the motivation to investigate the convergences of the tested segmental tunnel ring. This requires consideration of load case II, referring to rigid body displacements, associated with the relative rotation angles at the joints.

### 3.7 Load case II: structural analysis, considering the relative rotation angles at the joints

In load case II, the relative rotation angles that develop at the joints are considered. The point loads are set equal to zero. Following scientific research by Blom^18^ as well as by El Nagger and Hinchberger,^19^ it is assumed that the relative rotation angles at the joints result in *rigid body displacements* of the segments.

**Figure 17:**
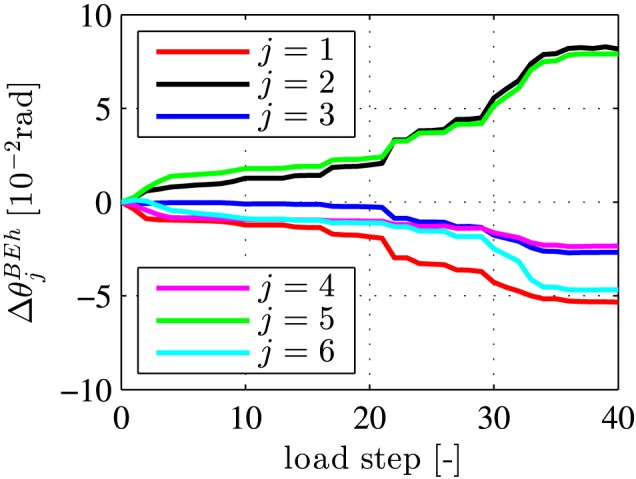
Relative rotation angles at joints, estimated from experimental monitoring data, based on the Bernoulli‐Euler hypothesis, see Equation 50 and Figures 4 and 5 [Colour figure can be viewed at wileyonlinelibrary.com]

First estimates of the relative rotation angles at the joints are based on the assumption of a *linear* distribution of circumferential displacement jumps along the radial length of the joints. In other words, the Bernoulli‐Euler hypothesis (index *B* *E* *h*) is used as a local kinematic hypothesis for the joints. Thus, the relative rotation angles are quantified as the difference of the circumferential displacement jumps, $\Delta v_{j}^{o}-\Delta v_{j}^{i}$, measured at the inner and the outer surface, see Figure 5, divided by the sensor distance *ℓ*_*s*_  =  410 mm, see Figure 4:

(50) $$\Delta \theta_{j}^{BEh}=\frac{\Delta v_{j}^{o}-\Delta v_{j}^{i}}{\ell_{s}}\;,\;j=1,2,\text{⋯},6\;,$$

see Figure 17 for the computed estimates of the relative rotation angles.

The estimated relative rotation angles do not relate to rigid body displacements only. This can be shown by means of the transfer relations, see (1). In each load step, only one transfer matrix is capable of describing the entire segmental tunnel ring. The last column of this transfer matrix includes six sets of load integrals (see Equations (9), (10), (11), (12)) corresponding to the six relative rotation angles according to Equation 50. Restricting the structural behavior to rigid body displacements of the segments, the ring must stay a closed ring (see Equations 14, 15, 16), and the static quantities at the crown, see Equations 17, 18, 19, must vanish: *M*_*i*_  =  *N*_*i*_  =  *V*_*i*_  =  0. Considering

(51) $$\sin(2\pi -\varphi_{j})=-\sin(\varphi_{j})\;,$$

(52) $$\cos(2\pi -\varphi_{j})=\cos(\varphi_{j})\;,$$

the following three conditions for rigid body displacements of the segments are obtained:

(53) $$\sum_{j=1}^{6}u^{L}(2\pi )=0\Rightarrow \sum_{j=1}^{6}\Delta \theta_{j}\sin(\varphi_{j})=0\;,$$

(54) $$\sum_{j=1}^{6}v^{L}(2\pi )=0\;\Rightarrow \;\sum_{j=1}^{6}\Delta \theta_{j}\left[1-\cos(\varphi_{j})\right]=0\;,$$

(55) $$\sum_{j=1}^{6}\theta^{L}(2\pi )=0\;\Rightarrow \;\sum_{j=1}^{6}\Delta \theta_{j}=0\;.$$

The estimates of the relative rotation angles according to Equation 50 (see also Figure 17) violate the conditions (53) to (55). Thus, these estimates do *not* refer to purely rigid body displacements of the segments. The underlying problem is the Bernoulli‐Euler hypothesis, which does not strictly apply to neck‐like joints. Thus, the estimates of the relative rotation angles need to be improved. Replacing the Bernoulli‐Euler hypothesis by a better *local* kinematic assumption for the neck‐like joints is, unfortunately, out of reach. As a remedy, the relative rotation angles are improved, based on considerations referring to the *global* scale of the entire segmental tunnel ring. Notably, this improvement is carried out in two steps.

The first improvement step refers to *symmetrization* of the relative rotation angles illustrated in Figure 17, such that

(56) $$\Delta \theta_{1}^{sym}=\Delta \theta_{6}^{sym}\;,$$

(57) $$\Delta \theta_{2}^{sym}=\Delta \theta_{5}^{sym}\;,$$

(58) $$\Delta \theta_{3}^{sym}=\Delta \theta_{4}^{sym}\;.$$

**Figure 18:**
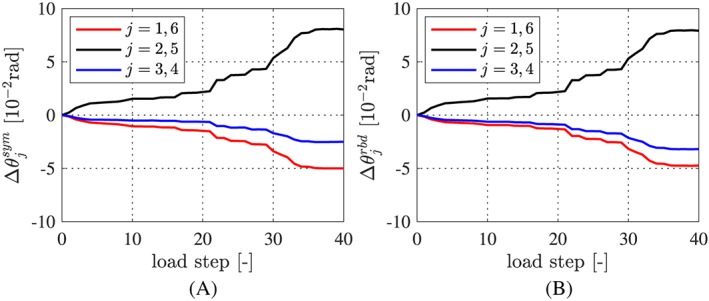
Improved estimates of the relative rotation angles at the joints: (A) symmetrized values computed according to Equation 59, based on the estimates illustrated in Figure 17, and (B) values referring to rigid body displacements of the segments, based on Equations 60 and (64), (65), (66) [Colour figure can be viewed at wileyonlinelibrary.com]

This is motivated by the fact that the external loading was symmetric with respect to the vertical axis of the ring and that the deformed configuration of the ring during the entire bearing‐capacity test was almost symmetric.^12^ Symmetrization is achieved by averaging the relative rotation angles, estimated for the two top joints, the two lateral joints, and the two bottom joints, respectively:

(59) $$\Delta \theta_{j}^{sym}=\frac{\Delta \theta_{j}^{BEh}+\Delta \theta_{7-j}^{BEh}}{2}\;,\;j=1,2,3\;,$$

see Figure 18A for the result. The symmetrized relative rotation angles satisfy the condition (53), but still violate the conditions (54) and (55). This raises the need for a second improvement step.

This improvement step refers to enforcing the conditions (54) and (55). Increments $\Delta \theta_{1}^{corr}$, $\Delta \theta_{2}^{corr}$, and $\Delta \theta_{3}^{corr}$ are added to the symmetrized values, $\Delta \theta_{1}^{sym}$, $\Delta \theta_{2}^{sym}$, and $\Delta \theta_{3}^{sym}$, in order to obtain relative rotation angles referring to rigid body displacements (index *r* *b* *d*). Thus,

(60) $$\Delta \theta_{j}^{rbd}=\Delta \theta_{j}^{sym}+\Delta \theta_{j}^{corr}\;,\;j=1,2,3\;,$$

where $\Delta \theta_{1}^{corr}$, $\Delta \theta_{2}^{corr}$, and $\Delta \theta_{3}^{corr}$ are unknown. Inserting the expressions (60) into the conditions (54) and (55) and considering Equations (56), (57), (58) yields, accounting for *φ*_*j*  =  1,2,…,6_  =  [8°,73°,138°,222°,287°,352°], the following two conditions for the three unknowns $\Delta \theta_{1}^{corr}$, $\Delta \theta_{2}^{corr}$, $\Delta \theta_{3}^{corr}$:

(61) $$\Delta \theta_{2}^{corr}=-\Delta \theta_{2}^{sym}-1.6740\left(\Delta \theta_{1}^{sym}+\Delta \theta_{1}^{corr}\right)\;,$$

(62) $$\Delta \theta_{3}^{corr}=-\Delta \theta_{3}^{sym}+0.6740\left(\Delta \theta_{1}^{sym}+\Delta \theta_{1}^{corr}\right)\;.$$

Equations 61 and 62 express $\Delta \theta_{2}^{corr}$ and $\Delta \theta_{3}^{corr}$ as functions of the *known* symmetrized relative rotation angles and of the *unknown* improved increment $\Delta \theta_{1}^{corr}$, which is the last remaining unknown. Equations 61 and 62 also underline that there are *infinitely many solutions* for $\Delta \theta_{1}^{corr}$, because there are infinitely many rigid body displacements that can be described by means of the improvement strategy expressed by Equation 60. Of these infinitely many solutions, it is reasonable to determine that solution which refers to the *smallest improved increments*. Herein, these increments are defined as the minimum of their sum of squares. Thus, the sought solution follows from the additional condition

(63) $$\frac{d\left[{\left(\Delta \theta_{1}^{corr}\right)}^{2}+{\left(\Delta \theta_{2}^{corr}\right)}^{2}+{\left(\Delta \theta_{3}^{corr}\right)}^{2}\right]}{d\Delta \theta_{1}^{corr}}=0\;.$$

Inserting the expressions for $\Delta \theta_{2}^{corr}$ and $\Delta \theta_{3}^{corr}$ according to Equations 61 and 62 into condition (63) delivers the sought result for $\Delta \theta_{1}^{corr}$ as

(64) $$\Delta \theta_{1}^{corr}=-0.7651\Delta \theta_{1}^{sym}-0.3933\Delta \theta_{2}^{sym}+0.1583\Delta \theta_{3}^{sym}\;.$$

Inserting this result into Equations 61 and 62 delivers the other two improved increments as

(65) $$\Delta \theta_{2}^{corr}=-0.3933\Delta \theta_{1}^{sym}-0.3417\Delta \theta_{2}^{sym}-0.2651\Delta \theta_{3}^{sym}$$

and

(66) $$\Delta \theta_{3}^{corr}=+0.1583\Delta \theta_{1}^{sym}-0.2651\Delta \theta_{2}^{sym}-0.8933\Delta \theta_{3}^{sym}\;.$$

The improved estimates of the relative rotation angles, which by definition refer to rigid body displacements, are quite similar to the symmetrized relative rotation angles (compare Figure 18A and B) and to the estimates obtained on the basis of the Bernoulli‐Euler hypothesis (compare Figure 18B with Figure 17).

The final estimates of the relative rotation angles of the joints are used to compute the rigid body displacements of the segments. As for every individual load step, this is achieved by means of a transfer matrix that includes six sets of load integrals, referring to the relative rotation angles of the six joints. In addition, *u*_*i*_  =  *v*_*i*_  =  *θ*_*i*_  =  *M*_*i*_  =  *N*_*i*_  =  *V*_*i*_  =  0. Thus, the solutions for the radial and circumferential displacement components follow from the first two lines of the transfer relations as

(67) $$u(\varphi )=-\sum_{j=1}^{6}R\Delta \theta_{j}^{rbd}\sin(\varphi -\varphi_{j})H(\varphi -\varphi_{j})\;,$$

(68) $$v(\varphi )=\sum_{j=1}^{6}R\Delta \theta_{j}^{rbd}\left[1-\cos(\varphi -\varphi_{j})\right]H(\varphi -\varphi_{j})\;.$$

Equations 67 and 68 describe rigid body displacements of the segments.

### 3.8 Superposition of load cases I and II: quantification of convergences

The solutions for the displacements due to load case I, “point loads,” and load case II, “relative rotation angles,” respectively, are used to compute the convergences of the analyzed segmental tunnel ring. They are equal to the absolute values of the changes of the diameter of the ring in the vertical and the horizontal direction. Thus, they can be computed based on the radial displacement component at the top and the bottom as well as at the “3 o'clock” position and the “9 o'clock” position, i.e. by

(69) $$c_{ver}=\left|u(0)+u(\pi )\right|\;,$$

(70) $$c_{hor}=\left|u(\frac{\pi }{2})+u(\frac{3\pi }{2})\right|\;.$$

**Figure 19:**
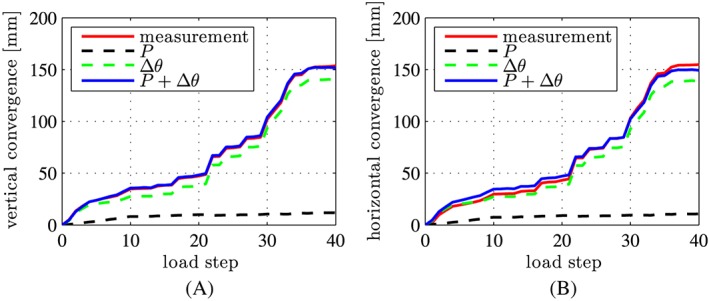
Comparison of the convergences obtained from simulation and measurements: (A) in the vertical direction; (B) in the horizontal direction [Colour figure can be viewed at wileyonlinelibrary.com]

The sum of the convergences computed in the two load cases agrees well with the experimental observations, compare the blue lines with the red lines in Figure 19. This underlines the usefulness of the chosen simulation strategy. Notably, the described agreement is not trivial, since the experimentally measured convergences are *not* used as input for the hybrid analysis and the model does *not* involve any fitted parameters.

Analyzing the contributions of the two load cases to the overall convergences shows that approximately 95% of the convergences refer to rigid body displacements that are related to the relative rotation angles at the joints (see the green lines in Figure 19) and that approximately only 5% result from deformations of the segments (see the black lines in Figure 19). Thus, the relative rotation angles at the joints govern the convergences of the analyzed segmental tunnel ring.

**Figure 20:**
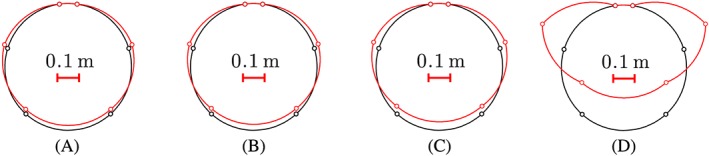
Results from nonlinear hybrid analysis: displacement of the axis of the ring at (A) load step 4, (B) load step 7, (C) load step 16, and (D) load step 40, associated with 25%, 50%, 75%, and 100% of the bearing capacity [Colour figure can be viewed at wileyonlinelibrary.com]

The displacement solutions of load case I, “point loads,” and load case II, “relative rotation angles,” are superimposed, resulting in the displacement of the axis of the analyzed segmental tunnel ring (see Figure 20). The displacement state strongly depends on the state of the relative rotation angles at the joints. The load increment from 0% to 25% of the bearing‐capacity results in considerable displacements (see Figure 20A) because the initial configuration—albeit being close to a perfect ring—had small geometric imperfections and the rigid body displacements of the segments, resulting from relative rotation angles at the joints, removed or, at least, reduced these imperfections significantly in the first 4 load steps.^13^ The load increments from 25% to 50% and 50% to 75% of the bearing‐capacity result in a rather small increase of the displacements (compare Figure 20B with Figure 20A and Figure 20C with Figure 20B). The load increment from 75% to 100% of the bearing‐capacity results in a significant increase of the displacements (compare Figure 20D with Figure 20C). By analogy, Figure 18 shows relatively large increments of rotation angles at the joints for load steps 0 to 4, small increments for load steps 4 to 7 and 7 to 16, and again relatively large increments for load steps 16 to 40. This corroborates the assertion that the relative rotation angles at the joints govern the state of displacements of the investigated segmental tunnel ring.

## 4 DISCUSSION

Two questions are posed at the beginning of the discussion. They read as follows: Are the relative rotation angles at the joints indeed related to rigid body displacements, or do they actually activate internal forces? How important is the consideration of bending‐induced tensile cracking of the segments in the context of quantifying the internal forces and the convergences?

### 4.1 Hybrid analysis under the assumption that the Bernoulli‐Euler hypothesis applies to neck‐like joints

Following Blom^18^ as well as El Nagger and Hinchberger,^19^ the present paper is based on the assumption that the relative rotation angles at the joints result in rigid body displacements of the segments. Accordingly, the relative rotation angles were determined in two steps. At first, they were estimated from the monitoring data, based on the Bernoulli‐Euler hypothesis. In the subsequent step, they were post‐processed in order to ensure that they result in rigid body displacements that do not activate internal forces in the ring. At this point, the necessity of the second post‐processing step is questioned.

This involves repetition of the nonlinear hybrid analysis of the bearing‐capacity tests, using two types of input: (1) the external forces and (2) the relative rotation angles, derived from the monitoring data based on the Bernoulli‐Euler hypothesis, see Equation 50. This time, the relative rotation angles also contribute to the internal forces of the ring. Given that tensile cracking of the segments renders the hybrid analysis nonlinear, a split of the analysis into two load cases is no longer admissible. Therefore, the external point loads and the relative rotation angles are considered simultaneously.

**Figure 21:**
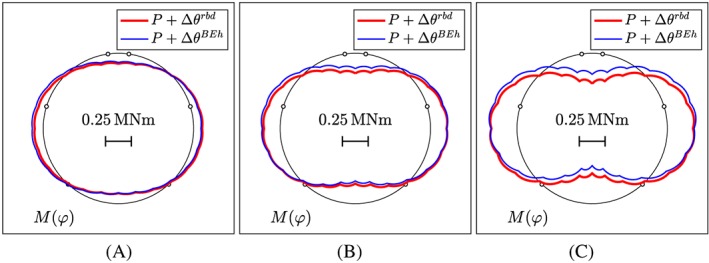
Comparison of simulation results from the two types of hybrid analyses: distributions of bending moments obtained at (A) load step 4, (B) load step 7, and (C) load step 16, associated with 25%, 50%, and 75% of the bearing capacity; the red lines refer to the hybrid analysis described in Section 3 and the blue lines to the hybrid analysis described in Section 4.1 [Colour figure can be viewed at wileyonlinelibrary.com]

**Figure 22:**
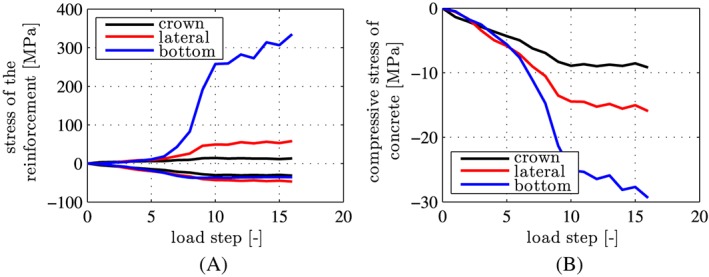
Results from simultaneous consideration of point loads and relative rotation angles $\Delta \theta_{j=1,2,\text{⋯},6}^{BEh}$; extreme values of computed stresses transferred by the segments in the top element #1, the lateral element #9, and the bottom element #17, as a function of the load step: (A) axial stress of the steel rebars, (B) compressive normal stress of concrete, in the circumferential direction [Colour figure can be viewed at wileyonlinelibrary.com]

The present hybrid analysis delivers *larger* internal forces of the segmental tunnel ring as compared with the analysis described in Section 3, see Figure 21 for bending moment distributions at load steps 4, 7, and 16, associated with 25%, 50%, and 75% of the bearing capacity, respectively. At load step 16, the performed hybrid analysis suggests that the steel reinforcement of segment ④ is so close to yielding in tension (see Figure 22) that continued loading up to load step 17 would exceed the elastic limit of the reinforcement. This result contradicts the experimental conclusion that the steel reinforcement of all segments remained linear elastic up to the bearing capacity of the ring.^12^

From the analysis, it is concluded that it is more realistic to assume that relative rotation angles result in rigid body displacements of the segments, as compared with the alternative approach of assuming that the Bernoulli‐Euler hypothesis applies to neck‐like joints between two neighboring segments. This corroborates the proposed mode of post‐processing the relative rotation angles such that they refer to rigid body displacements of the segments.

### 4.2 Can the internal forces and the convergences be quantified reliably, based on the assumption of linear‐elastic behavior of the segments?

The hybrid analysis described in Section 3 has shown that the convergences are governed by rigid body displacements of the segments, resulting from the relative rotation angles that develop at the joints. In the sense of an engineering mechanics approach, which aims at developing models that are as simple as possible and only as complex as necessary, it will be checked in the following, whether or not a linear‐elastic analysis of load case I, based on the average initial stiffnesses of the segments (Table 3), is sufficiently reliable for quantification of the internal forces and the convergences.

**Figure 23:**
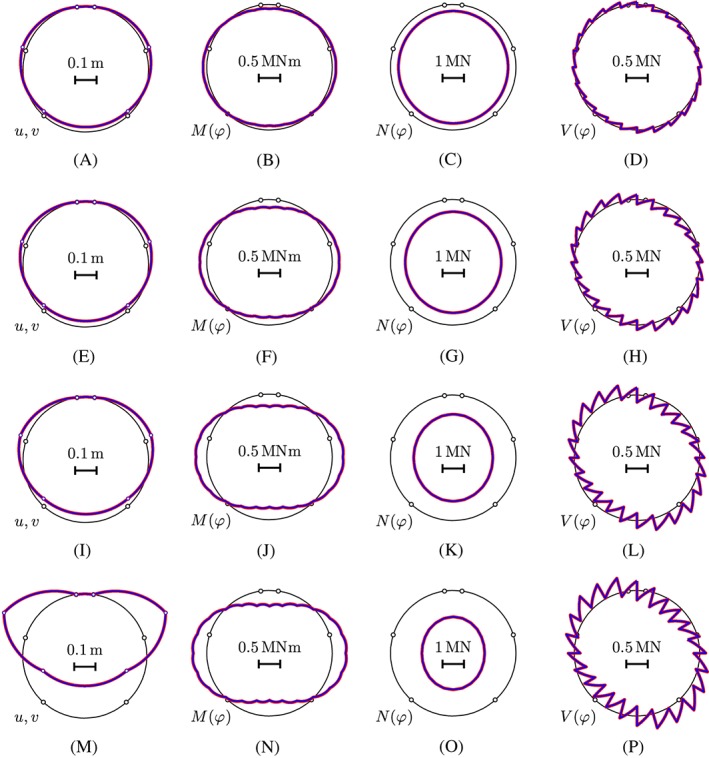
Comparison of displacements and internal forces computed, considering tensile cracking of the segments (see the red lines), and assuming linear‐elastic behavior of the segments (see the blue lines): (A‐D)  load step 4, (E‐H) load step 7, (I‐L) load step 16, and (M‐P) load step 40, associated with 25%, 50%, 75%, and 100% of the bearing capacity. As for the sign convention of the internal forces, the graphs located inside/outside of the axis of the segmental ring refer to negative/positive values [Colour figure can be viewed at wileyonlinelibrary.com]

The linear‐elastic version of load case I, “point loads,” refers to a continuous ring without joints, characterized by a uniform bending stiffness and a uniform extensional stiffness. The transfer relations (see Equations 1 and 2) are specified for 24 sets of load integrals (see Equations 3, 4, 5, 6, 7, 8) referring to the 24 point loads (see Figure 3). The static variables at the crown are determined, based on Equations (17), (18), (19). Results obtained from this analysis are compared with those from nonlinear structural analysis, described in Section 3.5, see Figure 23. The solutions obtained under the assumption of linear‐elastic behavior of the segments are virtually the same as the ones obtained for consideration of tensile cracking. The maximum difference regarding the internal forces at the bearing capacity of the ring (load step 40) amounts to 1.6%. This underlines that a linear‐elastic analysis is sufficient for estimation of the internal forces of the segmental tunnel ring. This agrees with the conclusion by Winkler et al.^36^ who found that tensile cracking of segments is of minor importance for the structural behavior of segmental tunnel linings.

## 5 CONCLUSIONS

The developed nonlinear hybrid method is well suited for multiscale analysis of displacement‐monitored segmental tunnel rings. It accounts for bending‐induced cracking of the segments and relative rotation angles at the joints, which refer to rigid body displacements of the segments. In the following, the conclusions drawn from this investigation are listed.

The developed nonlinear hybrid method requires the following minimum of input quantities:

• It requires knowledge of the external loading and the relative rotation angles that develop at the joints. The relative rotation angles are accessible from monitoring data of circumferential displacement discontinuities measured across the inner and the outer gaps of the joints in a real‐scale test. As for the design of tunnel linings, such monitoring data are unavailable. Therefore, the relative rotation angles must be computed by means of interface models, see Zhang et al.^20^
• Existing multiscale models allow for quantifying the stiffness, the uniaxial compressive strength, the tensile strength, and tensile softening of concrete, based on knowledge regarding the initial composition of the concrete of interest, defined by the mix design and the maturity of the material, typically quantified by means of hydration degrees.
• As regards the steel reinforcement of the segments, standard reinforcement drawings are required, as well as the stiffness and the yield stress of the steel.

Quantification of the relative rotation angles, developing at the joints during structural testing, is a nontrivial task.

• Quantifying relative rotation angles based on (1) monitoring data concerning displacement discontinuities in the circumferential direction, recorded across the joints both at the inner and the outer surface of the segments, and (2) the Bernoulli‐Euler hypothesis deliver first estimates only. They are not sufficiently reliable for structural analysis. The underlying reason is that the circumferential displacement discontinuities at the joints are actually *nonlinear* functions in the radial direction, because of stress concentrations in the neck‐like joint regions, bending‐induced separation of neighboring segments, and material nonlinearities, including compressive crushing of concrete and tensile yielding of steel bolts that are connecting neighboring segments.
• As long as the Bernoulli‐Euler hypothesis cannot be replaced by a better *local* kinematic assumption for the joints, it is recommended to improve the estimated relative rotation angles, at the *global* scale of the entire segmental tunnel ring, such that they result in rigid body displacements of all segments, as described in Section 3.7. In the context of the analyzed real‐scale test, this improvement included both symmetrization and the addition of the smallest possible correction increments.

The rigid body displacements resulting from the relative rotation angles govern the structural convergences and the bearing capacity of the tested segmental tunnel ring.

• As regards the analyzed real‐scale test, it was shown that rigid body displacements of the segments are responsible for approximately 95% of the convergences, while the deformation of the segments contributes to approximately 5% only. The latter contribution was estimated reliably with and without consideration of bending‐induced tensile cracking of the segments. As for practical purposes, it is, therefore, recommended to calculate the deformations of the segments under the assumption of linear‐elastic behavior, even if the tensile strength of concrete is exceeded in the simulations.
• The relative rotation angles governed the bearing capacity of the tested segmental tunnel ring, although the relative rotation angles at the joints were related, as a good approximation, to rigid body displacements of the segments. Thus, they had no significant influence on the internal forces of the segments. This can be explained as follows: The bearing capacity of the segmental tunnel ring is related to the development of a kinematic mechanism. The latter results from the development of plastic hinges at two pairs of joints, herein the two top joints and the two lateral joints.

As for estimating the internal forces experienced by the segments, structural analysis may be restricted to the external loading.

• Carrying out hybrid analyses with and without the assumption that relative rotation angles at the joints are associated with rigid body displacements of the segments, it was shown that these rotation angles are unlikely to result in significant internal forces of the segments. This conclusion is consistent with similar findings of Blom^18^ as well as of El Nagger and Hinchberger.^19^ Their findings were derived from the analysis of regular service loads, while a bearing‐capacity tests was analyzed in the present contribution.
• Carrying out hybrid analyses with and without consideration of bending‐induced tensile cracking, it was shown that the internal forces can be estimated reliably by means of *linear* hybrid analyses, disregarding tensile cracking of the segments. This conclusion is consistent with similar findings of Winkler et al.^36^

The presented mode of *nonlinear* analysis is nonetheless useful in the framework of durability analysis of segmental tunnel rings.

• When it comes to the design of reinforced concrete structures, the definition of serviceability limit states (SLS) includes limitations of the opening of cracks. These limitations ensure that the reinforcement is durably protected from corrosion‐inducing media. In the present analysis, crack openings were determined reliably, based on the employed multiscale model for quantification of the deterioration of the crack bands, as described in Sections 3.4 and 3.6.

The failure process of the joints governed the behavior of the analyzed segmental tunnel ring, from load step 4 to load step 40.

- The joints of the analyzed segmental tunnel ring can be described by *two* failure envelopes, representing *M*‐*N* interaction diagrams.
- The *inner* failure envelope refers to the largest resistance of the joints that can be established, based on the *initial contact area* between neighboring segments, see Figure 14. Once the inner failure envelope is reached, the corresponding joint behaves like a plastic hinge. The lateral joints and the top joints developed such plastic hinges already at load step 4 and load step 7, respectively. These load steps refer to intensities of the external loading, amounting to 25% and 50%, respectively, of the bearing capacity of the tested ring. It is concluded that some of the joints were “at failure” during 90 % of the load steps.
- Once the inner failure envelope is reached, relative rotation angles will increase significantly at almost constant loading. Adjacent to the compressed inner or outer surface of the joints, the large relative rotation will close the initial gap between the segments. Once segment‐to‐segment contact is established in this region, the joint will stiffen, as described in Liu et al.^7^
- The *outer* failure envelope refers to the maximum resistance of the joints. It can be established, based on both the initial contact area and the additional contact area, available after having reached the inner failure envelope, see Figure 15B. Once the outer failure envelope is reached, the joint has reached its bearing capacity and behaves like a plastic hinge. No further stiffening mechanism is possible.

It is recommended to organize structural analyses of segmental tunnel rings in form of the following six steps:

1. Analyze a continuous *linear‐elastic* ring subjected to the external loading in order to compute the internal forces and the structural displacements. This task can be accomplished comfortably, even in case of many point loads, with only one transfer matrix describing the entire ring structure.
2. As for quantification of crack‐opening displacements, which are relevant to durability design, determine the positions of the maximum bending moments and continue the analysis with the *nonlinear* model for crack bands as described in Sections 3.4 and 3.6.
3. Evaluate the internal forces at the position of the joints and use “interface models” to compute the relative rotation angles of the joints.
4. Post‐process the obtained relative rotation angles as described in Section 3.7 such that they refer to rigid body displacements of the segments.
5. Compute the structural displacements associated with the rigid body displacements of the segments, which result from the relative rotation angles at the joints. Again, this task can be accomplished comfortably, no matter how many joints have to be considered and where they are located, with only one transfer matrix, describing the entire ring structure.
6. Superimpose the displacements, obtained in steps 1 and 5, to obtain the total displacements of the segmental tunnel ring.

As long as realistic interface models are not available, the combination of displacement‐monitoring data and hybrid analysis remains a prime candidate for structural analysis of segmental tunnel rings, loaded all the way up to their bearing capacity.

## APPENDIX A AN ESTIMATION OF THE VOLUME FRACTIONS OF THE PHASES OF THE CONSIDERED CONCRETE

The bearing‐capacity test was carried out 28 days after casting of the segments. Herein, the corresponding hydration degrees of the cement, the slag, and the fly ash are estimated as *ξ*_*c**e**m**e**n**t*_  =  0.8,^37^ *ξ*_*s**l**a**g*_  =  0.4,^38^ and *ξ*_*f**l**y**a**s**h*_  =  0, respectively. Starting from scale I (see Figure 7), the volume fractions of solid C‐S‐H and gel pore are estimated, based on Termkhajornkit's experiments,^37^ as

(A1) $$f_{solidCSH}^{I}=0.6\;\;\text{and}\;\;f_{gelpore}^{I}=0.4\;.$$

As for scales II and III, the estimation of the volume fractions of the compositions begins with their volumes. The volumes of clinker, fly ash, and slag are calculated based on their hydration degree:

(A2) $$V_{clinker}=\frac{m_{cement}}{\rho_{cement}}(1-\xi_{cement})\;,$$

(A3) $$V_{flyash}=\frac{m_{flyash}}{\rho_{flyash}}(1-\xi_{flyash})\;,$$

(A4) $$V_{slag}=\frac{m_{slag}}{\rho_{slag}}(1-\xi_{slag})\;.$$

The volume of the hydrates (i.e. C‐S‐H gel at scale II) follows from two chemical reactions: hydration of cement and of slag. Powers' hydration model of cement^39^ delivers the volume fractions of compositions with respect to the cement paste. Multiplying these fractions by the sum of the initial volume of cement and water gives their volumes, resulting from hydration of cement:

(A5) $$V_{H_{2}O}^{cp}=\frac{63(w/c-0.42\xi_{cement})}{20+63w/c}\cdot \left(\frac{m_{cement}}{\rho_{cement}}+\frac{m_{H_{2}O}}{\rho_{H_{2}O}}\right)\;,$$

(A6) $$V_{hyd}^{cp}=\frac{43.15\xi_{cement}}{20+63w/c}\cdot \left(\frac{m_{cement}}{\rho_{cement}}+\frac{m_{H_{2}O}}{\rho_{H_{2}O}}\right)\;,$$

(A7) $$V_{void}^{cp}=\frac{3.31\xi_{cement}}{20+63w/c}\cdot \left(\frac{m_{cement}}{\rho_{cement}}+\frac{m_{H_{2}O}}{\rho_{H_{2}O}}\right)\;,$$

where *w*/*c* is the initial water‐to‐cement mass ratio. These volumes are corrected by considering the hydration of slag, based on Hlobil's experimental investigation of the volume fractions of blended cement paste.^40^ According to his results, one volume unit of slag typically reacts with the same volume of water and produces two volume units of hydrates. Therefore, the volumes of the *consumed* water and of the *produced* hydrates by hydration of slag is equal to one time and two times, respectively, of the volume of the hydrated slag, i.e.

(A8) $$V_{H_{2}O}^{slag}=\frac{m_{slag}}{\rho_{slag}}\xi_{slag}\;,$$

(A9) $$V_{hyd}^{slag}=2\;\frac{m_{slag}}{\rho_{slag}}\xi_{slag}\;.$$

Combining the hydration of cement and of the slag delivers the volumes of the individual phases as follows:

(A10) $$V_{H_{2}O}=V_{H_{2}O}^{cp}-V_{H_{2}O}^{slag}\;,$$

(A11) $$V_{hyd}=V_{hyd}^{cp}+V_{hyd}^{slag}\;,$$

(A12) $$V_{void}=V_{void}^{cp}\;.$$

Then the volume fractions of the individual phases can be calculated by the following formulae:

(A13) $$f_{CSHgel}^{II}=\frac{V_{hyd}}{V_{hyd}+V_{void}+V_{H_{2}O}},$$

(A14) $$f_{H_{2}O}^{II}=\frac{V_{H_{2}O}}{V_{hyd}+V_{void}+V_{H_{2}O}},$$

(A15) $$f_{void}^{II}=\frac{V_{void}}{V_{hyd}+V_{void}+V_{H_{2}O}},$$

(A16) $$f_{hyd.foam}^{III}=\frac{V_{hyd}+V_{H_{2}O}+V_{void}}{\frac{m_{cement}}{\rho_{cement}}+\frac{m_{flyash}}{\rho_{flyash}}+\frac{m_{slag}}{\rho_{slag}}+\frac{m_{H_{2}O}}{\rho_{H_{2}O}}},$$

(A17) $$f_{clinker}^{III}=\frac{V_{clinker}}{\frac{m_{cement}}{\rho_{cement}}+\frac{m_{flyash}}{\rho_{flyash}}+\frac{m_{slag}}{\rho_{slag}}+\frac{m_{H_{2}O}}{\rho_{H_{2}O}}},$$

(A18) $$f_{slag}^{III}=\frac{V_{slag}}{\frac{m_{cement}}{\rho_{cement}}+\frac{m_{flyash}}{\rho_{flyash}}+\frac{m_{slag}}{\rho_{slag}}+\frac{m_{H_{2}O}}{\rho_{H_{2}O}}},$$

(A19) $$f_{flyash}^{III}=\frac{V_{flyash}}{\frac{m_{cement}}{\rho_{cement}}+\frac{m_{flyash}}{\rho_{flyash}}+\frac{m_{slag}}{\rho_{slag}}+\frac{m_{H_{2}O}}{\rho_{H_{2}O}}}.$$

As for the scales IV and V, the volume fractions follow from the initial composition of the concrete, reading as

(A20) $$f_{sand}^{IV}=\frac{\frac{m_{sand}}{\rho_{sand}}}{\frac{m_{cement}}{\rho_{cement}}+\frac{m_{flyash}}{\rho_{flyash}}+\frac{m_{slag}}{\rho_{slag}}+\frac{m_{H_{2}O}}{\rho_{H_{2}O}}+\frac{m_{sand}}{\rho_{sand}}}\;,$$

(A21) $$f_{agg}^{V}=\frac{\frac{m_{agg}}{\rho_{agg}}}{\frac{m_{cement}}{\rho_{cement}}+\frac{m_{flyash}}{\rho_{flyash}}+\frac{m_{slag}}{\rho_{slag}}+\frac{m_{H_{2}O}}{\rho_{H_{2}O}}+\frac{m_{sand}}{\rho_{sand}}+\frac{m_{agg}}{\rho_{agg}}}\;.$$

## APPENDIX B M‐N INTERACTION DIAGRAMS FOR THE JOINTS

### B.1 Mathematical details of the first failure envelope

Considering the stress distribution illustrated in Figure 14, the resulting normal force *N* and the bending moment *M* are given as

(B1) $$|N|=F\;f_{c}\;b\;x-f_{b}\;A_{b}\;,$$

(B2) $$|M|=F\;f_{c}\;b\;x\left(\frac{h}{2}-\frac{x}{2}\right)+f_{b}\;A_{b}\;e_{b}\;,$$

where *b*  =  1.0 m and *h*  =  0.24 m stand for the width and the thickness, respectively, of the initial contact area. Further, *A*_*b*_ and *e*_*b*_ denote the area and the eccentricity of the bolts and *x* stands for the length of the compression zone of concrete, see Figure 14. *F* is computed as^20^

(B3) $$F=\sqrt{\frac{3\;B}{b}}\;.$$

The interaction envelope is obtained by solving Equation B1 for *x* and inserting the resulting expression into Equation B2. This gives

(B4) $$|M|=(|N|+F\;f_{b}\;A_{b})\left(\frac{H}{2}-\frac{|N|+f_{b}\;A_{s}}{2\;F\;f_{c}\;B}\right)+f_{b}\;A_{b}\;e_{b}.$$

This formula can be used to predict the development of plastic hinges at the joints, described by the first failure envelope.

### B.2 Mathematical details of the second failure envelope

Considering the stress distribution illustrated in Figure 15B, the resulting normal force *N* and the bending moment *M* are given as

(B5) $$|N|=f_{c}\;B\;x-f_{b}\;A_{b}\;,$$

(B6) $$|M|=f_{c}\;B\;x\left(\frac{H}{2}-\frac{x}{2}\right)+f_{b}\;A_{b}\;e_{b}\;.$$

The interaction envelope is obtained by solving Equation B5 for *x* and inserting the resulting expression into Equation B6. This yields

(B7) $$|M|=(|N|+f_{b}\;A_{b})\left(\frac{H}{2}-\frac{|N|+f_{b}\;A_{s}}{2\;f_{c}\;B}\right)+f_{b}\;A_{b}\;e_{b}.$$

This formula can be used to predict the bearing capacity of the joints.
